# Enhancing microtubule stabilization rescues cognitive deficits and ameliorates pathological phenotype in an amyloidogenic Alzheimer’s disease model

**DOI:** 10.1038/s41598-020-71767-4

**Published:** 2020-09-08

**Authors:** Juan Jose Fernandez-Valenzuela, Raquel Sanchez-Varo, Clara Muñoz-Castro, Vanessa De Castro, Elisabeth Sanchez-Mejias, Victoria Navarro, Sebastian Jimenez, Cristina Nuñez-Diaz, Angela Gomez-Arboledas, Ines Moreno-Gonzalez, Marisa Vizuete, Jose Carlos Davila, Javier Vitorica, Antonia Gutierrez

**Affiliations:** 1 Dpto. Biología Celular, Genética y Fisiología, Instituto de Investigación Biomédica de Málaga-IBIMA, Facultad de Ciencias, Universidad de Málaga, Campus de Teatinos, 29071 Málaga, Spain; 2 Centro de Investigación Biomédica en Red sobre Enfermedades Neurodegenerativas (CIBERNED), Madrid, Spain; 3 Dpto. Bioquímica y Biología Molecular, Facultad de Farmacia, Universidad de Sevilla, C/Prof. Garcia Gonzalez 2, 41012 Sevilla, Spain; 4 Instituto de Biomedicina de Sevilla (IBIS), Hospital Universitario Virgen del Rocio/CSIC, Universidad de Sevilla, Sevilla, Spain

**Keywords:** Cellular neuroscience, Diseases of the nervous system

## Abstract

In Alzheimer’s disease (AD), and other tauopathies, microtubule destabilization compromises axonal and synaptic integrity contributing to neurodegeneration. These diseases are characterized by the intracellular accumulation of hyperphosphorylated tau leading to neurofibrillary pathology. AD brains also accumulate amyloid-beta (Aβ) deposits. However, the effect of microtubule stabilizing agents on Aβ pathology has not been assessed so far. Here we have evaluated the impact of the brain-penetrant microtubule-stabilizing agent Epothilone D (EpoD) in an amyloidogenic model of AD. Three-month-old APP/PS1 mice, before the pathology onset, were weekly injected with EpoD for 3 months. Treated mice showed significant decrease in the phospho-tau levels and, more interesting, in the intracellular and extracellular hippocampal Aβ accumulation, including the soluble oligomeric forms. Moreover, a significant cognitive improvement and amelioration of the synaptic and neuritic pathology was found. Remarkably, EpoD exerted a neuroprotective effect on SOM-interneurons, a highly AD-vulnerable GABAergic subpopulation. Therefore, our results suggested that EpoD improved microtubule dynamics and axonal transport in an AD-like context, reducing tau and Aβ levels and promoting neuronal and cognitive protection. These results underline the existence of a crosstalk between cytoskeleton pathology and the two major AD protein lesions. Therefore, microtubule stabilizers could be considered therapeutic agents to slow the progression of both tau and Aβ pathology.

## Introduction

In Alzheimer’s disease (AD), and other neurodegenerative tauopathies, tau protein is abnormally hyperphosphorylated and aggregated into intraneuronal neurofibrillary tangles (NFTs)^1^. AD is also characterized by the presence of extracellular amyloid plaques, composed predominately of amyloid-β (Aβ) peptides, along with microglial response and neuroinflammation, axonal/synaptic damage and neuronal loss^2–7^.

The structural integrity and function of axonal microtubules (MT) depends on the microtubule-associated protein tau^8,9^. Since neurons are extremely polarized cells from both a morphological and functional point of view, they largely depend on transport along their axons to be completely healthy and well-functioning^10,11^, which in turn relies on the proper binding of tau to tubulins^12^. Significantly, a number of axonal-flow dependent mechanisms have been reported to be disturbed in neurons from AD patients and models^13–16^, which exhibit intra/extracellular Aβ accumulation, intraneuronal aberrant aggregates of hyperphosphorylated (phospho)-tau, dystrophic neurites and interferences in anterograde/retrograde transport. Therefore, it is not surprising that both gain-of-toxicity and loss-of-function of tau, together with the consequent microtubular destabilization, have arisen as novel putative pharmacological targets^17–20^. Thus, MT-targeting agents are attractive therapeutic candidates for neurodegenerative diseases^20,21^. In fact, different MT-stabilizing compounds like taxanes or epothilones, used in anticancer chemotherapy, have been recently assessed in some preclinical studies using transgenic models of tauopathy^22,23^. Specifically, Epothilone D (EpoD) is a brain-penetrant drug that has arisen as a good candidate to test the potential of microtubule stabilizers as a treatment for neurodegenerative diseases. In fact, several drugs that affect microtubule stability are currently under investigation as potential treatments for AD^18,20^. However, as far as we are concerned, to date the assessment of MT-stabilizing compounds has not been evaluated in amyloidogenic AD models and the impact of this therapeutic approach on amyloid-associated pathology remains to be dilucidated.

In this work we have evaluated the effect of intraperitoneal administration of EpoD in a double-transgenic mouse model, APP/PS1, starting before the onset of the pathology (from 3 to 6 months of age). The hippocampus of these mice exhibits significant amyloid extracellular deposits which are decorated with axonal/synaptic dystrophic neurites^24–31^. Moreover, this model develops an important GABAergic neuronal loss at early ages in hippocampus, entorhinal and perirhinal cortices^30–35^. Though these mice do not reproduce the formation of NFTs, they develop pathological posttranslational modifications of tau leading to AT8-positive aggregates within dystrophic neurites showing a connection between Aβ and tau pathology. Our data indicated that microtubule stabilization induced by EpoD treatment promoted memory improvement, synaptic/neuritic recovery and neuronal protection. Furthermore, EpoD treatment reduced not only tau pathology, but more interestingly, the intra- and extracellular Aβ accumulation. Thus, MT-stabilizing compounds may be used for targeting both Aβ and tau pathology in AD treatment.

## Results

### EpoD rescues memory deficits in APP/PS1 mice

**Figure 1:**
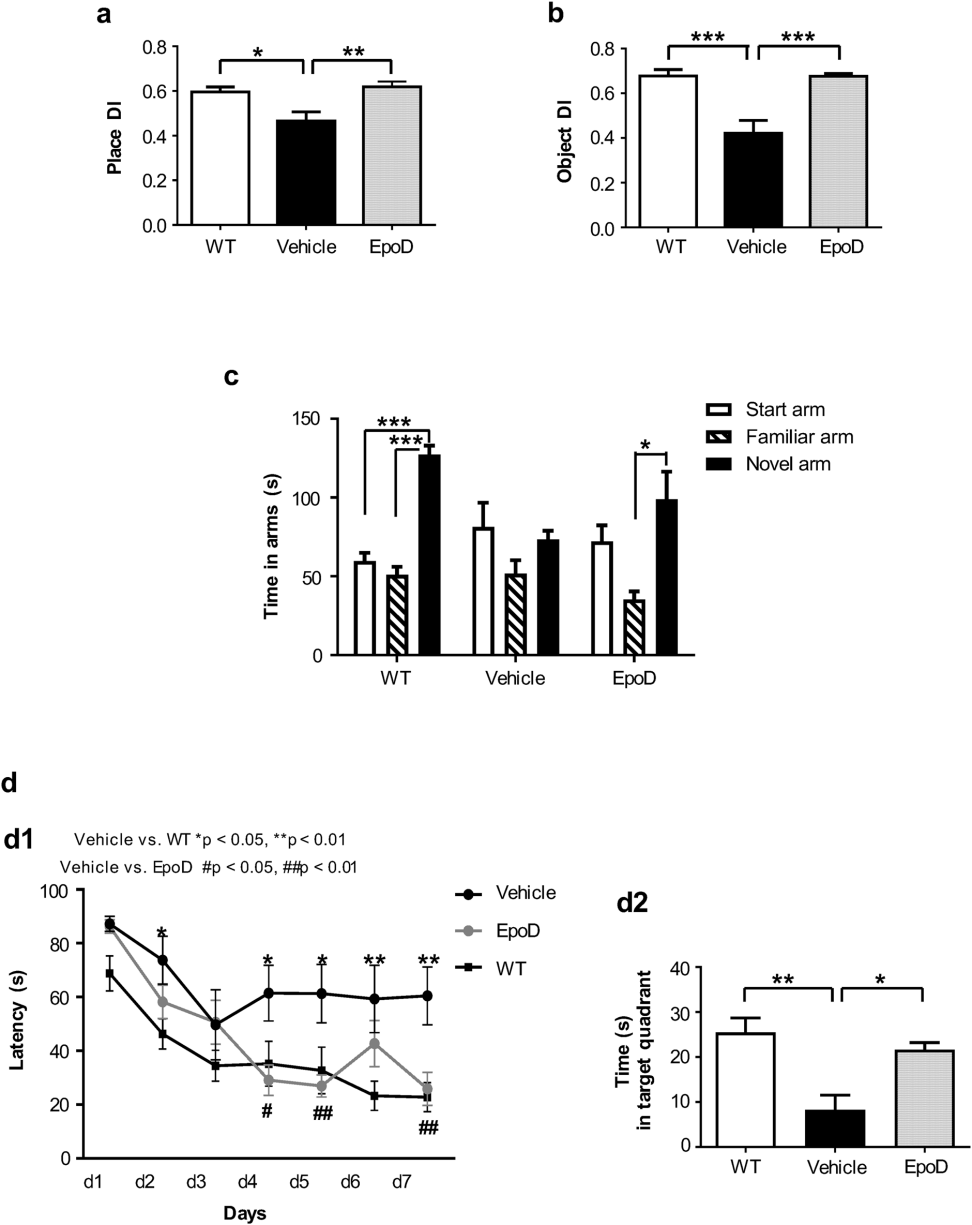
EpoD treatment improved cognitive function in APP/PS1 mice. (**a**) At old-location test, one-way ANOVA (F(2,22) = 7.842, p = 0.003) revealed significant differences between groups in place discrimination index (place DI). Post-hoc analysis showed that task performance of APP/PS1^Veh^ animals (Tukey, *p < 0.05, **p < 0.01) exhibited significant differences compared with WT or APP/PS1^EpoD^ mice. (**b**) Similarly, at novel object recognition task, one-way ANOVA (F(2,22) = 16.47, p < 0.0001) unveiled significant differences between groups in object DI. Post-hoc analysis showed a significant better performance in EpoD animals or WT mice, in comparison to vehicle (Tukey, ***p < 0.001). (**c**) Time spent in each arm during the retention phase of the Y-maze specific context test. One-way ANOVA of repeated measures (RM) was performed for each group. No arm preferences were found in APP/PS1^Veh^ mice (F(2,10) = 3.075, p = 0.13), in contrast to EpoD (F(2,14) = 5.15, p = 0.046) and WT (F(2,18) = 41.933, p = 0.0001) groups. Post-hoc analysis determined that both APP/PS1^EpoD^ (Tukey, *p < 0.05) and WT (Tukey, ***p < 0.0001), but not the vehicle group, spent more time in the novel than in the familiar arm (time in novel arm F(2,21) = 5.56, p = 0.011). (**d**) Spatial learning and memory were evaluated in the Morris Water Maze using a 7-day hidden platform acquisition (4 trials/day). (**d1**) All groups decreased scape latency over the training. Two-way ANOVA revealed significant differences along learning days (F(6,126) = 23.05, p < 0.001) and between groups (F(2,126) = 5.81, p < 0.01). In addition, there was a significant interaction between the treatment and the latency (F(12,126) = 2.138, p = 0.019). Post-hoc analysis showed poorer learning of vehicle animals as compared with either EpoD or WT groups (Tukey, EpoD vs. vehicle ^#^p < 0.05, ^##^p < 0.01; WT vs. vehicle *p < 0.05, **p < 0.01). No differences were found between APP/PS1^EpoD^ and WT. (**d2**) In the probe test, one-way ANOVA (F(2,20) = 9.34, p = 0.014) showed that both APP/PS1^EpoD^ and WT exhibited similar preference for the target quadrant in contrast to vehicle transgenic mice. Post-hoc testing determined that APP/PS1^Veh^ spent less time swimming in the target quadrant than EpoD and WT groups (Tukey, *p < 0.05, **p < 0.01, respectively). *s* seconds. All data shown correspond to mean ± SEM.

We first tested whether EpoD treatment prevents/ameliorates cognitive deficiencies in our amyloidogenic model. APP/PS1^EpoD^ and APP/PS1^Veh^ mice were evaluated the last 2 weeks of treatment. Non-treated age-matched WT mice were also included for comparison purposes. Open-field test (OFT) was performed to determine baseline activity as well as to evaluate whether EpoD treatment or vehicle injections provokes alterations on the locomotor and exploratory behavior. No significant differences between the three groups were observed for either, the distance roamed, speed or the number of rearings (Supplemental Fig. S1). Therefore, APP/PS1^EpoD^ or APP/PS1^Veh^ mice did not display locomotive or exploratory behavior alterations when compared to age-matched WT animals. We next evaluated the effects of EpoD on episodic-like memory using the old-location task (OLT, spatial memory) and the novel object recognition task (NORT, recognition memory), utilizing the natural tendency of the animals to preferentially explore novel objects or placed in new locations (spontaneous exploratory behavior). OLT data (Fig. 1a) revealed that EpoD treatment protected from the cognitive deficits observed in vehicle-injected APP/PS1 group (p < 0.01). In fact, APP/PS1^EpoD^ mice performed indistinguishable from WT mice. Similarly, NORT data (Fig. 1b) indicated that APP/PS1^Veh^ mice performed worse than APP/PS1^EpoD^ group (p < 0.001), which in turn behaved similarly to WT mice, demonstrating a protective action of this compound on this type of memory.

Long-term memory was analyzed using a variant of the Y-maze with patterned walls (Fig. 1c) to evaluate whether mice are able to distinguish the maze arms according to the time spent in each arm during the retention phase. APP/PS1^EpoD^ mice exhibited a significant preference for the novel arm compared to the familiar one (p < 0.05), as the WT mice did (p < 0.0001), in contrast to vehicle-injected mice which did not discriminate between novel and familiar arm. No significant differences in distance or speed were observed among groups (Supplemental Fig. S1).

The effect of EpoD treatment on learning and memory was also evaluated by Morris Water Maze (MWM; Fig. 1d). Navigational learning and memory skills were assessed using a 7-day hidden platform acquisition (4 trials/day). No visual defects were detected with visible platform task among groups (not shown). During acquisition, all groups learned to locate the hidden platform by spatial navigation, as demonstrated by decreases in latency (Fig. 1d1). Importantly, APP/PS1^EpoD^ mice needed less time to find the scape platform than the APP/PS1^Veh^ group (p < 0.01), performing very similar to WT mice. Therefore, these results indicated that EpoD treatment was involved in the preservation of APP/PS1 allocentric learning. Afterwards, during the probe trial (retention phase) (Fig. 1d2), APP/PS1^Veh^ mice displayed memory deficits, spending significant less time in the target zone (p < 0.05). However, no differences were observed between APP/PS1^EpoD^ and WT mice, suggesting that EpoD treatment was also able to keep the short-term spatial memory ability in APP/PS1 mice.

Taken together, all these data indicated that EpoD treatment prevented the onset of hippocampal-related cognitive impairment in APP/PS1 mice.

### EpoD improves microtubular stability in the hippocampus of APP/PS1

Next, to evaluate the microtubule-stabilizing effect of EpoD we analyzed the expression of acetylated tubulin (AcTub), a well-known biomarker of stable microtubules^36^. Tubulin acetylation takes place after polymerization into microtubules, and the most stable pools are known to be enriched in AcTub^37,38^. Immunofluorescence analysis of this marker by confocal microscopy evidenced a moderate/intense neuronal cell bodies, apical dendrites and axonal tracts in the hippocampus of WT mice (CA1 region and alveus are depicted in Supplemental Fig. S2). APP/PS1^Veh^ mice exhibited lower AcTub immunoreactivity than WT mice, indicating a higher microtubule instability. Interestingly, a stronger immunostaining for AcTub was observed in APP/PS1^EpoD^ mice in comparison to the vehicle group, indicating a better preservation of microtubule integrity in the hippocampal neurons of the EpoD group.

### EpoD protects against Aβ-induced synaptic/axonal pathology

**Figure 2:**
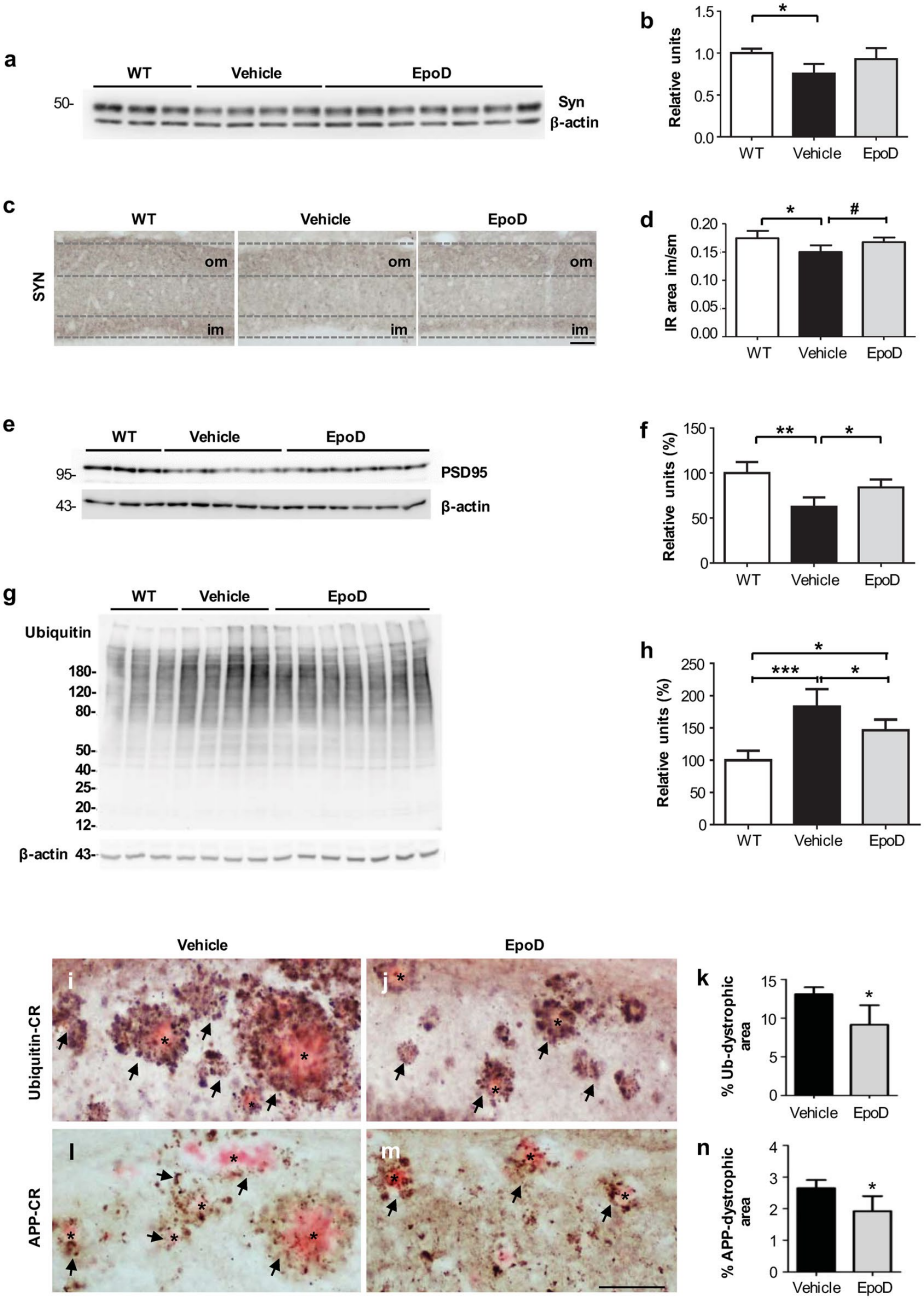
EpoD treatment reduced both synaptic and plaque-associated neuritic pathology. (**a**,**b**) Representative western blot and quantitative analysis of synaptophysin (Syn) in total hippocampal proteins from WT (n = 3), vehicle (n = 4) and EpoD (n = 7) mice. For quantification, data were referred to WT group. β-Actin was used as the loading control. Syn protein levels were decreased in vehicle group and recovered in EpoD mice (ANOVA F(2,11) = 4.386, p = 0.039, Tukey, *p < 0.05). (**c**) Syn-immunostaining shows a laminar pattern in the molecular layer of the hippocampal dentate gyrus. (**d**) The area occupied by the Syn-positive inner band of the molecular layer (im) in APP/PS1^EpoD^ animals was similar to that in WT mice, and significant bigger than in APP/PS1^Veh^ mice (ANOVA F(2,12) = 5.644, p = 0.019, Tukey, *p < 0.05). (**e**,**f**) Representative western blot and quantitative analysis of the postsynaptic marker PSD95 in total hippocampal proteins from WT, vehicle and EpoD mice. For quantification, data were referred to WT group. β-Actin was used as the loading control. PSD95 protein levels were significantly recovered in EpoD group (ANOVA (F (2,10) = 12.44, p = 0.0019, Tukey, *p < 0.05). (**g**,**h**) Quantitative western blot for ubiquitin (ANOVA F(2,11) = 14.97, p < 0.001) showed a significant increase in vehicle mice (Tukey, ***p < 0.001) and a significant reduction (Tukey, *p < 0.05) in the hippocampus of treated mice as compared with WT. It is noteworthy that EpoD reverted partially (as compared with WT, Tukey, *p < 0.05) the ubiquitinated protein accumulation. Expression of the different markers were always referred to WT. Hippocampal sections were immunolabeled for ubiquitin (**i**,**j**) or APP (**l**,**m**), and counterstained with Congo red (CR), to evidence protein build-up within dystrophic neurites around amyloid plaques (asterisk, see details in insets). Quantitative image analysis demonstrated a significant decrease of (**k**) ubiquitin- (*t* test, *p < 0.05) and (**n**) APP-positive area (*t* test, *p < 0.05) in CA1 subfield in APP/PS1^EpoD^ in comparison to controls. Uncropped blots are shown in Supplementary Fig. S6. *im* inner molecular layer, *om* outer molecular layer, *slm* stratum lacunosum-moleculare, *so* stratum oriens, *sp* stratum pyramidale, *sr* stratum radiatum. Scale bars: (**c**) 50 μm; (**i**,**j**) and (**l**,**m**) 100 μm.

As we have previously reported, the hippocampus of the APP/PS1 model develops prominent axonal/presynaptic pathology (plaque-associated dystrophic neurites formation) since early ages^27–32,34^. The functional integrity of axons/synapses depends on the axonal transport and, consequently, on microtubules. Thus, aiming to investigate whether the protection against the cognitive decline observed in APP/PS1^EpoD^ mice was due to a synaptic/axonal preservation, we analyzed the expression of synaptophysin (Syn) and PSD95, classic presynaptic and postsynaptic markers respectively. Western blot analysis (Fig. 2a,b) showed a significant decrease of Syn levels in APP/PS1^Veh^ mice (75.40 ± 11.68% vs. WT mice, p < 0.05), which was almost recovered by EpoD treatment (92.86 ± 13.19% vs. WT mice; 1.23-fold increase respect to vehicle group). Next, we validated these data by immunostaining (Fig. 2c). Typically, in WT mice the outer and the inner layers of the dentate gyrus stratum moleculare showed a higher Syn immunoreactivity than the middle one (Fig. 2c), according to the spatial arrangements of entorhino-dentate projections^39^ together with local GABAergic afferents^40^. However, Syn-immunostaining (Fig. 2c,d) within the inner molecular layer in APP/PS1^Veh^ dentate gyrus was reduced (− 14.28 ± 6.9% vs. WT mice, p < 0.05) whereas it was preserved in APP/PS1^EpoD^ mice (+ 10.28 ± 5.53% vs. vehicle group, p < 0.05). Accordingly, levels of the postsynaptic marker PSD95, measured by western blots (Fig. 2e,f), were significantly higher (1.34-fold) in the hippocampus of EpoD-treated mice compared to vehicle group (84.01 ± 8.80% and 62.46 ± 10.44%, respectively; data compared to WT mice. EpoD vs. vehicle p < 0.05; WT vs. vehicle p < 0.01).

This synapse protection could be a consequence of EpoD-mediated amelioration of the previously reported axonal pathology in this model^28,29^. Thus, we next assessed the accumulation of total ubiquitinated proteins and the ubiquitin-positive dystrophies in the hippocampus (Fig. 2g–k). Quantitative western blotting (Fig. 2g,h) revealed that the levels of ubiquitinated proteins were indeed increased in APP/PS1^Veh^ mice (182.83 ± 27.38%, p < 0.001 as compared with WT animals) and consequently reduced in APP/PS1^EpoD^ hippocampus (146.42 ± 16.54%, p < 0.05 as compared with vehicle). These data were confirmed by ubiquitin immunolabeling combined with image analysis. As previously reported and showed in Fig. 2i,j, ubiquitin immunostaining accumulated in dystrophic neurites surrounding Aβ plaques. EpoD treatment produced a significant reduction (− 30.10 ± 19.18%, p < 0.05) in the CA1 ubiquitin loading in APP/PS1^EpoD^ animals, compared to APP/PS1^Veh^ (Fig. 2k). Since dystrophies were also positive for APP-protein^28^, the EpoD-dependent reduction in dystrophic neurites was also corroborated using an anti-APP antibody. As shown (Fig. 2l–n), we observed a significant reduction (− 27.51 ± 18.12%, p < 0.05) in the APP-loading surrounding Aβ plaques.

Therefore, EpoD treatment preserved the synaptic integrity, reduced the accumulation of ubiquitinated proteins and decreased the dystrophic pathology in this amyloidogenic model. All these neuroprotective effects should reflect an amelioration of the axonal transport defects by improving microtubular stability.

### Microtubular stabilization reduces SOM interneuron loss

**Figure 3:**
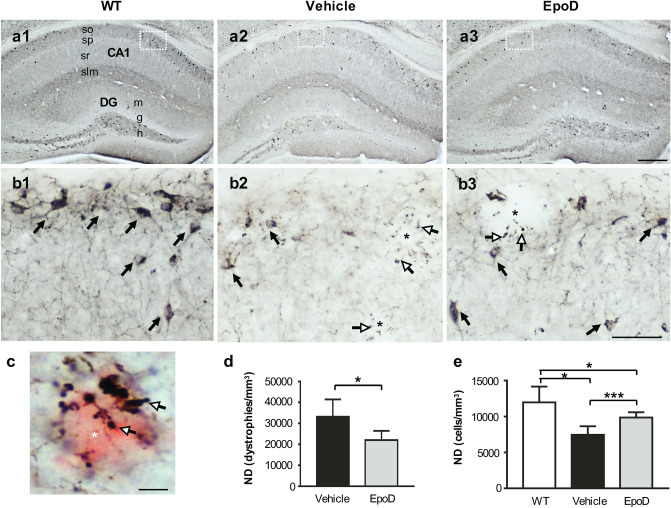
Epothilone D treatment ameliorated hippocampal SOM-interneuron degeneration. (**a1**–**a3**) Panoramic view of SOM-immunolabeled hippocampal sections of WT (**a1**), APP/PS1^Veh^ (**a2**) and APP/PS1^EpoD^ (**a3**) mice. (**b1**–**b3**) Higher magnification of the stratum oriens from the CA1 subfield showing a reduction in the amount of SOM-positive somata (black arrows) and the corresponding neuropile in vehicle group (**b2**) in contrast to EpoD (**b3**) and WT (**b1**). (**b2**,**b3**) SOM-positive dystrophies in APP/PS1 mice were also evidenced (white arrows, neuritic dystrophies; asterisks, amyloid plaques). (**c**) As visualized by Congo red counterstaining, SOM-immunoreactive dystrophic neurites (white arrows) in APP/PS1 mice are associated with amyloid plaques (red). (**d**,**e**) Numerical densities (ND) of SOM-positive dystrophies and neurons in CA1 subfield were assessed by stereology. WT (n = 4), APP/PS1^Veh^ (n = 4) and APP/PS1^EpoD^ (n = 7). (**d**) As expected, EpoD provoked a significant decrease in the amount of SOM-positive aberrant neurites in this region (EpoD vs. vehicle, *t* test, *p < 0.05). No SOM-positive dystrophies were observed in WT mice. (**e**) Quantitative analysis demonstrated a significant loss of SOM-interneurons in APP/PS1^Veh^ and a partial neuroprotective effect in EpoD-treated group as compared with WT (ANOVA, F(2,12) = 11.15; p = 0.0018; Tukey, EpoD vs. vehicle ***p < 0.001; vehicle vs. WT *p < 0.05). *CA* cornu Ammonis, *DG* dentate gyrus, *h* hilus, *g* granular layer, *m* stratum moleculare, *slm* stratum lacunosum-moleculare, *so* stratum oriens, *sp* stratum pyramidale, *sr* stratum radiatum. Scale bars: (**a1**–**a3**) 250 μm; (**b1**–**b3**) 50 μm; (**c**) 10 μm.

A significant loss of GABAergic SOM interneurons in memory-relevant brain regions is an early pathological event in this APP/PS1 model^31,33–35^. Therefore, we assessed the density of SOM-cells and SOM-positive dystrophic neurites in the CA1 subfield of hippocampus by stereological quantification (Fig. 3). As expected, APP/PS1^Veh^ mice (Fig. 3a2,b2) showed abundant SOM-positive dystrophic neurites around amyloid plaques (Fig. 3c), and displayed a significant decrease (− 37.57 ± 9.8%, p < 0.05) of SOM-positive neurons (Fig. 3e), compared to age-matched WT mice (Fig. 3a1,b1). Noteworthy, EpoD treatment (Fig. 3a3,b3) significantly reduced the amount of plaque-associated SOM-positive dystrophies (− 33.63 ± 13.17%, p < 0.05, compared to vehicle group; Fig. 3d). More relevant, the numerical density (cells/mm^3^) of SOM neurons was higher in APP/PS1^EpoD^ (+ 20.07 ± 9.73%, p < 0.001) than in vehicle group, though did not reach WT values (Fig. 3e). Thus, these data suggested that stabilization of microtubules by EpoD was able to protect, at least in part, this vulnerable hippocampal interneuron subpopulation not only from axonal damage but also from cell death in this APP/PS1 model.

### EpoD reduces both phospho-tau and Aβ pathology

**Figure 4:**
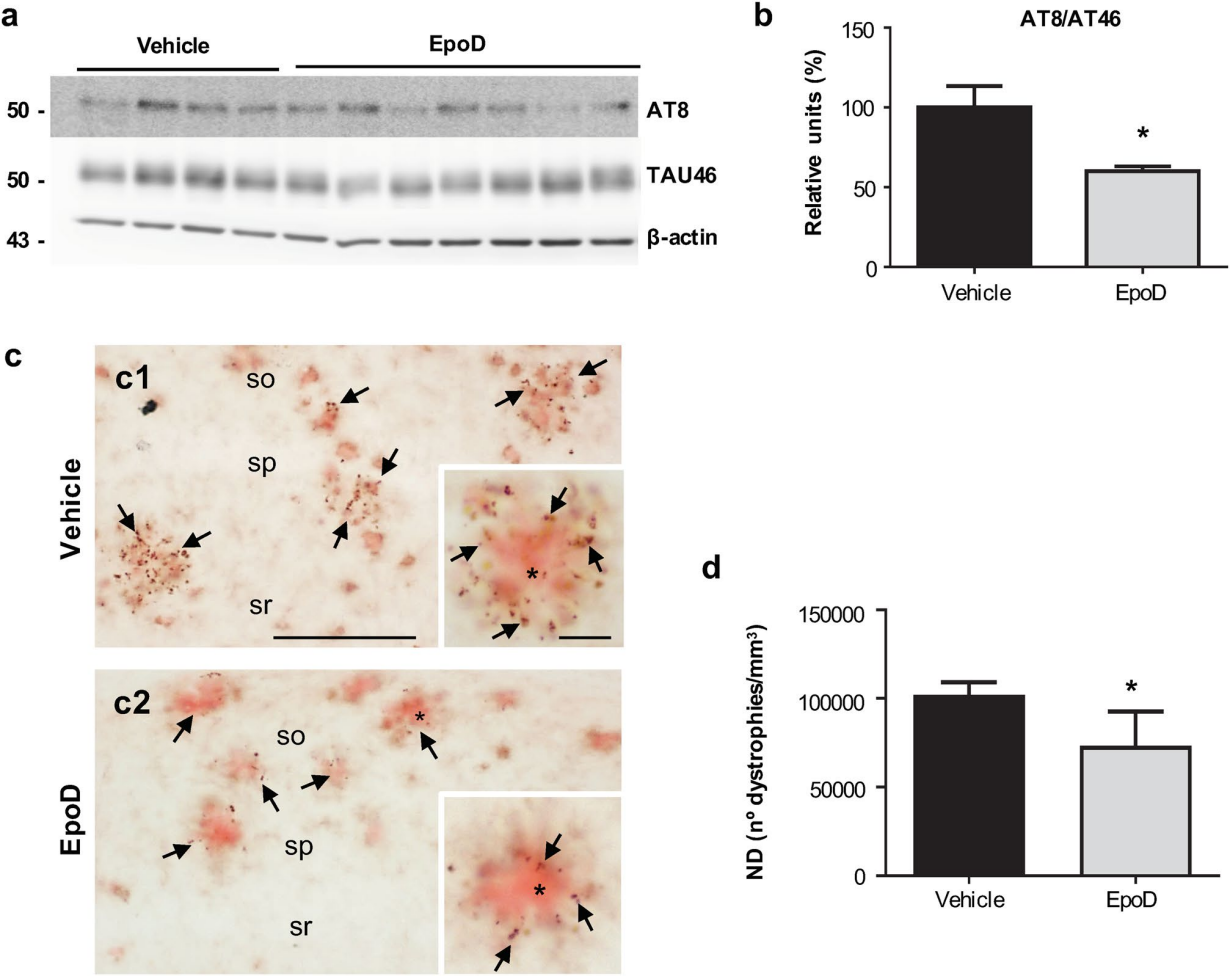
Tau pathology decreased in the hippocampus of EpoD-treated mice. (**a**,**b**) Representative western blot and quantitative analysis of the phospho-tau ratio (AT8/TAU46) from APP/PS1^Veh^ (n = 4) and APP/PS1^EpoD^ (n = 7) mice. β-Actin was used as the loading control. For quantification, ratio was referred to vehicle group. The ratio of phospho-tau/total tau was significantly reduced in treated mice (*t* test, *p < 0.05). Uncropped blots are shown in Supplementary Fig. S7. (**c**) AT8-positive dystrophies (arrows) were located surrounding Aβ plaques (Congo red, asterisk). Quantitative analysis (**d**) showed a significant decrease in the numerical density (ND) of AT8-positive dystrophic neurites (*t* test, *p < 0.05) in CA1 region from EpoD (**c2**) compared to vehicle mice (**c1**). *so* stratum oriens, *sp* stratum pyramidale, *sr* stratum radiatum. Scale bars: (**c1**) and (**c2**) 200 μm; insets 20 μm.

To determine the impact of EpoD treatment on the two major AD-pathological lesions we evaluated phospho-tau and Aβ accumulation in the hippocampus. This APP/PS1 model exhibits phospho-tau build-up within dystrophic neurites surrounding amyloid plaques^24,28,29^. Thus, we first assessed the AT8/TAU46 ratio by western blot (Fig. 4a,b). As expected, there was a clear accumulation of AT8-positive tau in APP/PS1^Veh^ mice that was significantly reduced after EpoD treatment (60.07 ± 6.70% vs. 100 ± 26.89%, for EpoD or vehicle mice, respectively, p < 0.05). This decrease in phospho-tau levels was paralleled by a reduction on AT8-positive dystrophic neurites (− 27.17 ± 19.1%, p < 0.05) around amyloid deposits in hippocampal CA1 region after EpoD treatment (Fig. 4c,d). Hence, these data indicate that EpoD treatment attenuated tau pathology.

**Figure 5:**
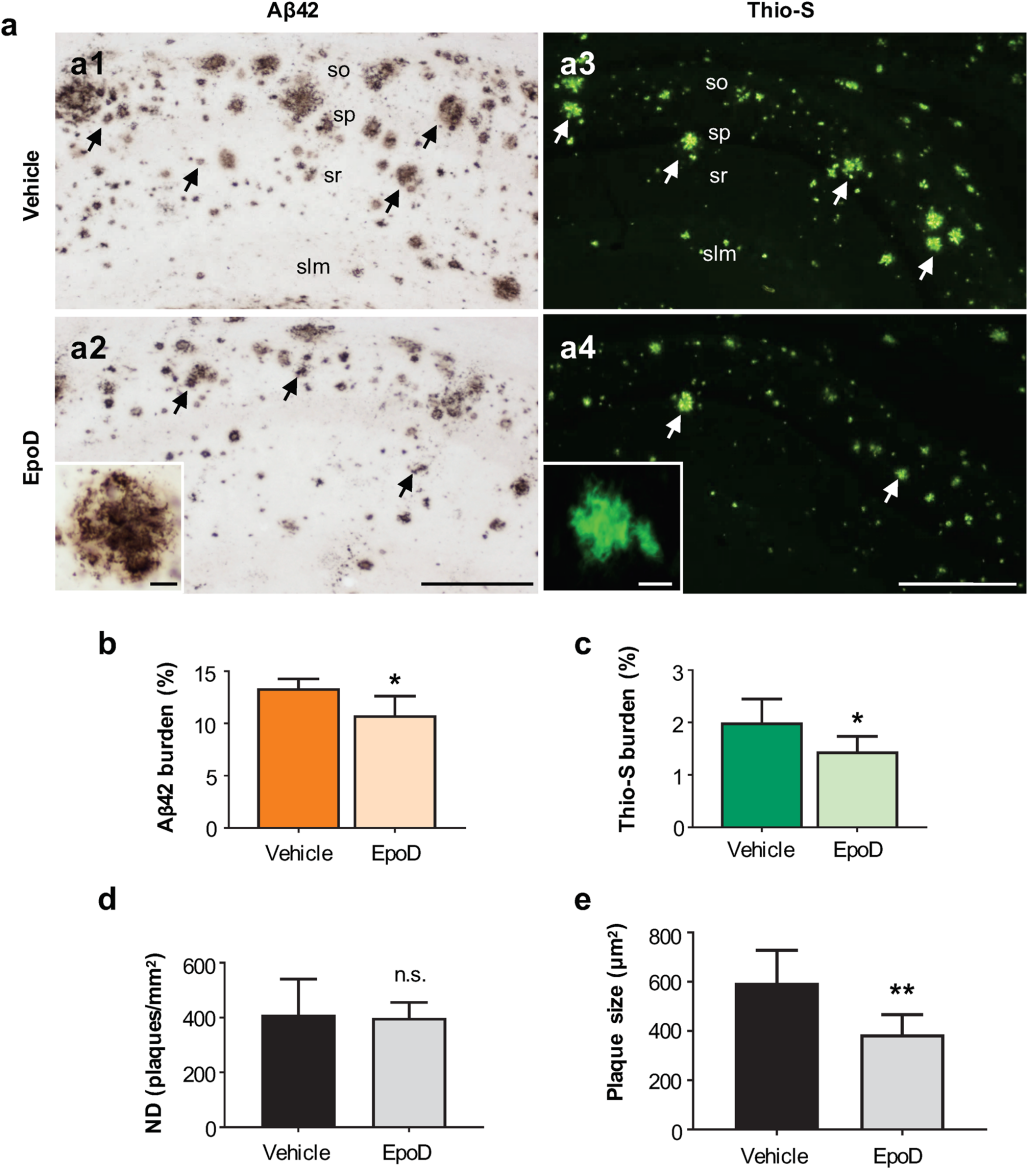
EpoD treatment reduced Aβ pathology in APP/PS1 hippocampus. (**a**) Extracellular Aβ accumulation was assessed by both Aβ42 immunohistochemistry (**a1**,**a2**) and Thioflavin-S staining (**a3**,**a4**) for fibrillary plaques in the CA1 subfield. Insets show higher magnifications of stained amyloid plaques. (**b**) Quantitative analyses demonstrated a significant reduction of Aβ42 deposition in CA1 region (*t* test, *p < 0.05) of EpoD mice (n = 7) in comparison to vehicle group (n = 4). (c) Similarly, Thio-S loading was also found to be reduced in the hippocampus of EpoD treated mice (*t* test, *p < 0.05). (**d**) The numerical density (ND) of plaques did not change, but plaque size (**e**) was significantly smaller in EpoD group (*t* test, **p < 0.01). *slm* stratum lacunosum-moleculare, *so* stratum oriens, *sp* stratum pyramidale, *sr* stratum radiatum. Scale bars: (**a1**–**a4**) 200 μm; insets 20 μm.

**Figure 6:**
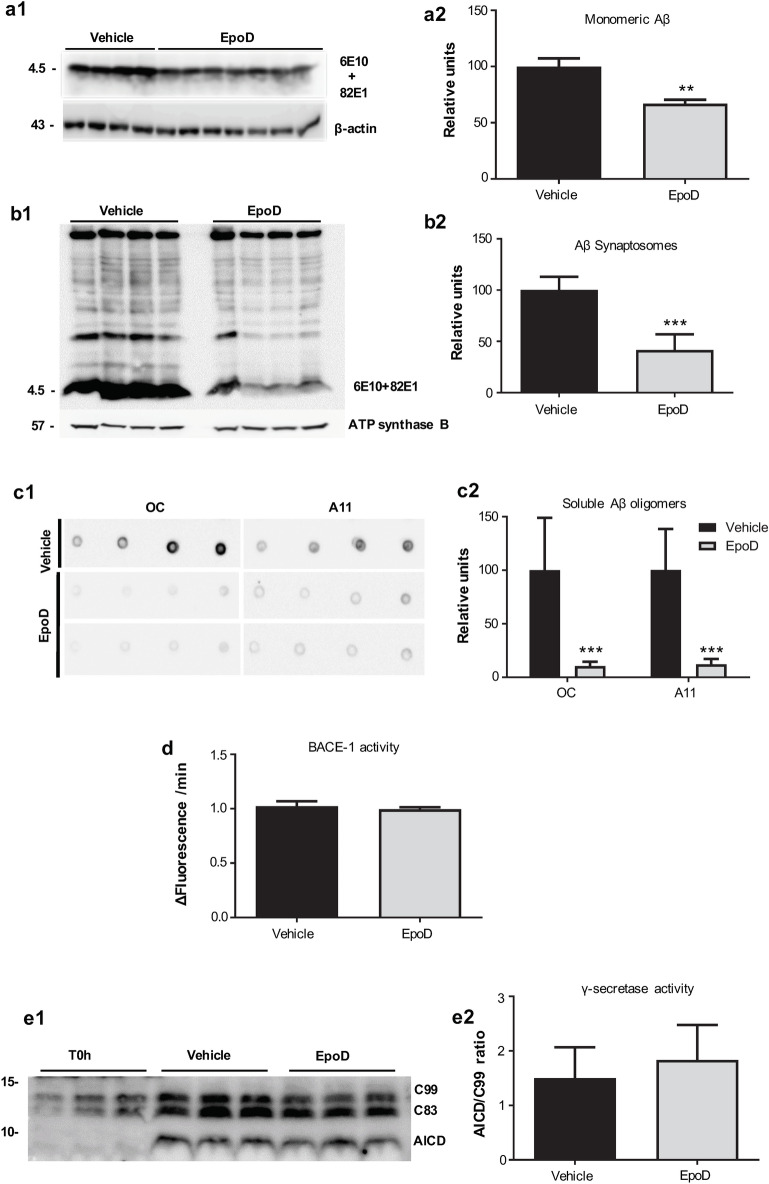
Effect of EpoD treatment on the Aβ production. (**a1**,**a2**) Quantitative western blots using 6E10 plus 82E1 antibodies in total proteins isolated from hippocampus of vehicle (n = 4) and EpoD mice (n = 7). APP/PS1^EpoD^ animals showed a significant reduction of monomeric Aβ levels as compared with the vehicle group (*t* test, **p < 0.01). For quantification, levels were referred to vehicle group. (**b1**,**b2**) Importantly, western blotting of isolated hippocampal synaptosomes using the same antibodies demonstrated a stronger reduction in Aβ content in this neuronal fraction of EpoD mice (*t* test, ***p < 0.001; n = 4 mice/group). (**c1**,**c2**) EpoD treatment also produced a strong reduction (***p < 0.001) on the soluble oligomeric Aβ accumulation. Aβ oligomers were assayed using OC or A11 dot blots. Equivalent S1 proteins from WT was used as negative controls. Protein loading was controlled using parallel dot-blots developed using ant-GAPDH (Supplemental Fig. S4). Direct effect of EpoD on BACE-1 (**d**) and γ-secretase (**e1**,**e2**) activities. No significant differences were observed (n = 6 independent experiments). Uncropped blots are shown in Supplementary Fig. S9.

Next, we analyzed the extracellular Aβ accumulation in the CA1 subfield by Aβ42 immunohistochemistry (Fig. 5a1,a2; hippocampal panoramic images are shown in Supplemental Fig. S3) as well as using the conformation-dependent dye Thioflavin-S for fibrillar amyloid species (Fig. 5a3,a4). With both approaches (Fig. 5b,c), extracellular Aβ burden was found to be slightly but significantly diminished after the EpoD treatment (− 19.5 ± 17.02% for Aβ42 and − 27.75 ± 20.06% for Thio-S, p < 0.05). Stereological quantification of plaque numerical density (plaques/mm^2^) in CA1 region (Fig. 5d) demonstrated that the density of amyloid deposits did not significantly change after treatment, however, their size (Fig. 5e) was significantly smaller (− 35.07 ± 13.46%, p < 0.01) in APP/PS1^EpoD^ group compared to vehicle mice. Furthermore, this decrease was concomitant with a reduction in hippocampal total monomeric Aβ levels in APP/PS1^EpoD^ compared to APP/PS1^Veh^ mice (66.93 ± 9.40% vs. 100 ± 14.69%, p < 0.01) measured by western blot (Fig. 6a1,a2).

In order to further investigate this effect on Aβ production, we next analyzed the intracellular Aβ accumulation on synaptosomes isolated from Vehicle and EpoD-treated APP/PS1 mice (Fig. 6b1,b2). We have previously demonstrated^29^ that Aβ accumulates in the synaptosomal fraction and this accumulation could reflect the axonal pathology in this model. Thus, the microtubule/axonal stabilization could reduce the Aβ content within the presynaptic terminals. As shown (Fig. 6b1,b2), EpoD-treated mice exhibited a striking reduction on synaptosomal Aβ content compared to vehicle group (41.62 ± 15.35% vs. 100 ± 12.91%, for EpoD or vehicle groups, respectively p < 0.001). We next evaluated whether this decrease in the total or synaptosomal Aβ accumulation was also reflected by a reduction on the soluble oligomeric Aβ forms. To test this possibility, the S1 soluble fractions were isolated and the Aβ oligomers were assayed using anti-oligomeric OC and A11 antibodies in dot blot experiments (Fig. 6c1,c2). As expected from previous works, S1 fractions isolated from APP/PS1^Veh^ mice accumulated both OC- and A11-positive Aβ oligomers. These Aβ oligomeric forms were highly reduced (18.41 ± 6.25% vs. 100.00 ± 47.35%, p = 0.0006; 14.18 ± 4.22% vs. 100.30 ± 23.62, p = 0.0001 for OC and A11 in EpoD or vehicle groups, respectively) in S1 fractions isolated from EpoD treated APP/PS1 mice.

Aiming to confirm that microtubule stabilization could indeed reduce the Aβ production, we have treated N2a-APP cell line with EpoD and the intracellular Aβ content was tested by western blots. Even using this in vitro system and an acute treatment (12 h or 24 h of treatment, Supplemental Fig. S5 a1 and a2), EpoD produced a significant reduction on the intracellular Aβ in N2a-APP cell line (41.63 ± 4.92% or 48.71 ± 4.27% vs. 100.01 ± 23.71% for 12 h and 24 h EpoD vs. Control, respectively; p < 0.05). It is also noteworthy that APP-CTFs were not significantly altered under these experimental conditions (Supplemental Fig. S5 d1 and d2). Thus, even under in vitro conditions, EpoD produced an appreciable effect on Aβ production.

These data could be explained by a direct inhibitory effect of EpoD on either BACE-1 or γ-secretase activities. Therefore, we tested by in vitro assays the possible inhibitory effect of EpoD on both APP-processing enzymatic activities. As shown (Fig. 6d), BACE-1 activity was not directly affected by EpoD treatment. The absence of BACE-1 inhibition is also consistent with the absence of modifications in the soluble APPβ or in C99-CTF fragments (Supplemental Fig. S5 c1, c2 and d1, d2, respectively). Similarly, we observed no changes on soluble APPα or C83-CTFs fragments, indicating the absence of α-secretase inhibition (Supplemental Fig. S5 c1, c2 and d1, d2, respectively). Moreover, as shown in Fig. 6e1,e2, EpoD had not any appreciable direct effect on γ-secretase activity.

Together, these data revealed that microtubule stabilization by EpoD administration reduced in APP/PS1 mice the synaptic pathology, dystrophic neurites formation, SOM pathology and Aβ accumulation, producing a preservation of hippocampal-dependent memory. EpoD also reduced the Aβ production in an amyloidogenic cell model. This effect on Aβ pathology was not mediated by altering BACE1 or γ-secretase activities.

## Discussion

Many neurodegenerative pathologies, including Alzheimer’s disease (AD), share microtubule (MT) abnormalities and axonal transport disruption^15,18^. Consequently, MT-targeting agents are attractive therapeutic candidates for these devastating brain diseases^20^ and several clinical trials to evaluate microtubule stabilizers efficacy are currently ongoing^21^. MT-stabilizing drugs have been previously tested in Tau transgenic mouse models resulting in improved axonal transport and motor function^41,42^. However, the impact of MT-stabilizers on Aβ pathology has not been assessed in vivo yet.

To the best of our knowledge, this is the first preclinical study using a brain-penetrant MT-stabilizer in an amyloidogenic transgenic model of Alzheimer’s disease. We demonstrate that early administration of EpoD prevented cognitive decline and ameliorated AD-like pathology in the hippocampus of APP/PS1 mice. Besides the expected beneficial impact on tau pathology, EpoD treatment reduced the extracellular and presynaptic Aβ accumulation. Moreover, EpoD administration decreases axonal/synaptic damage, dystrophic neurites formation and neuronal loss. Therefore, our data support that destabilization of cytoskeleton integrity is implicated in the progression of Aβ pathology and neuronal vulnerability.

Microtubules are critical for neuronal function and survival^10,43^. Post-translational modifications of tubulin are involved in the regulation of microtubule dynamics, constituting a useful code of functional information^44,45^. For instance, the most stable pools, long-lived microtubules, are enriched in acetylated tubulins which protect them from mechanical aging^37,38^. A reduction of acetylated tubulin levels has been reported in AD brains^46^. In addition, it has been shown that Aβ increases MT instability^47^. In agreement with these observations, the expression of acetylated α-tubulin was low in our transgenic APP/PS1 mice, indicating that unstable and depolymerized MTs may also contribute to AD pathology in this amyloidogenic model. Importantly, EpoD treatment resulted in an enrichment of acetylated tubulin and thus in the amount of stable MTs, as reported previously in models of tauopathy and Parkinson’s disease^48–50^. Then, this could translate into improved MT function and axonal transport in EpoD mice, and consequently, lead to reduced AD-pathology and clinical manifestations.

In line with this, the peripheral administration of EpoD, at the initial stages of the pathology, prevented the hippocampal-related cognitive deficits displayed by this amyloidogenic transgenic model. Similarly, EpoD treatment has proven to produce cognitive recovery in tauopathy models^42,48,50^. It is well established that synaptic damage, induced by soluble Aβ oligomers, is the best correlate of cognitive deficiencies in early stages of AD^51,52^, and our APP/PS1 model displays hippocampal synaptic alterations since early ages (this work; see also^28^). Therefore, EpoD treatment may promote cognitive function improvement in our model by preserving the synaptic integrity through microtubular stabilization, either directly enhancing the intracellular trafficking of protein cargos, or indirectly via a reduction of Aβ production or accumulation. EpoD seems to act both pre- and post-synaptically, increasing the levels of both synaptophysin and PSD95 proteins, respectively, and this effect could be probably due to the improvement of the microtubular-dependent traffic in both axonal and dendritic compartments. In this sense, it has been shown that EpoD reverses the Aβ-induced spine loss in organotypic cultures from APP hippocampus^53^. Also, an increase of the synaptic density was found in the CA3 subfield after EpoD treatment in a tauopathy model^50^. On the other hand, it has been demonstrated that during synapse elimination in the neuromuscular junction, the axonal branches dismantle their microtubules, and that the injection of Epothilone B is enough to delay this process^54^, highlighting the involvement of microtubular stabilization in synaptic maintenance. We have previously demonstrated that most of dystrophic neurites in APP/PS1 mice correspond to aberrant axons/presynaptic terminals^27,28,31^, which are characterized by cytoskeletal defects^29^ and accumulation of Aβ peptides^28,29,31^. Interestingly, after EpoD treatment we detected a significant decrease in the dystrophic neurite number, the accumulation of ubiquitinated proteins, and the presynaptic Aβ content. Therefore, the restauration of microtubular integrity, together with the reduction of phospho-tau, probably ameliorate the axoplasmic flow and the synaptic preservation. Thus, it is highly likely that the synaptic recovery, linked to the reduction of axonal pathology, might explain the behavioral improvement after the EpoD treatment.

In addition, the close spatiotemporal relationship between all dystrophic neurites and amyloid plaques^55^ indicates that there is a direct connection between both pathological events. In fact, Aβ is able to disrupt MTs^47,56,57^, thus promoting dystrophies formation, and dystrophies could be a source of Aβ production^28,29,58^, leading to a self-perpetuating cycle. Here, we found that EpoD treated mice show a significant reduction of both intracellular and extracellular Aβ content in the hippocampus. Moreover, the treatment induced a reduction in plaque size. We have confirmed this effect of EpoD on Aβ accumulation using an independent Alzheimer’s model. EpoD-treated N2a-APP cell cultures resulted in significant lower Aβ levels. However, the β- and γ-secretase activities were not altered by EpoD. Therefore, the decrease in Aβ intracellular content, in the absence of C99 levels modification, might be explained by an EpoD mediated effect on intracellular vesicular transport, decreasing the probability of C99 and γ-secretase subcellular co-localization at synapses, and in consequence, decreasing the intracellular Aβ production^59^. Alternatively, EpoD-mediated MT stabilization, by improving the axonal transport, may increase the Aβ degradation at the lysosomal compartment. Both circumstances agree with the drastic reduction on the synaptosomal Aβ content, and the soluble Aβ oligomeric forms, observed in this work after EpoD treatment.

A relevant finding of this study is the preservation of the highly vulnerable somatostatin-expressing interneurons in the EpoD treated animals. This neuronal population is extensively damaged in the APP/PS1 mouse, and the loss of somatostatin neurons has been closely associated to the Aβ burden^30,31,33–35^. In this regard, it is important to mention that, in this specific APP/PS1 model, only principal neurons are able to express the mutated human transgene^28,34,35^, and thus the SOM pathology is induced by the extracellular Aβ. Therefore, the effect of EpoD on the interneurons survival could be indirectly mediated by the Aβ diminution, or interneuron preservation could also reflect an improvement in axonal transport, reduced intracellular protein accumulation and synaptic protection. Present results do not allow us to discriminate if the EpoD-protective effect is due to the reduction on extracellular Aβ accumulation or the amelioration of MT instability. Probably both effects, reduction on plaques size together with diminution on the SOM-positive dystrophies, are implicated on the protection of this vulnerable population. To the best of our knowledge, this is the first report showing that EpoD prevents neuronal loss in an in vivo model of amyloidosis. EpoD-mediated SOM cell protection could explain, at least in part, the cognitive recovery in treated mice. GABAergic dysfunction has been postulated as major contributor for the cognitive impairment manifested by AD patients^60^. SOM neuron loss is a well-established pathogenic event in AD brains^35,61–63^. Considering that SOM participates in synaptic plasticity and memory process^64–67^, the loss of these interneurons may contribute to the cognitive deficits associated to AD. In addition, the EpoD-induced SOM protection may also contribute to the reduced amyloid pathology observed in the treatment group mice. SOM regulates the levels of the Aβ-cleaving enzyme neprilysin^68^, interferes with the Aβ fibrillization^69^ and regulates the Aβ-induced blood–brain barrier permeability^70^, indicating that the loss of this GABAergic subpopulation plays a pivotal role in AD pathogenesis.

In sum, our results indicated that either microtubule stabilization or the indirect effects induced by EpoD treatment promoted spatial memory, synaptic/neuritic recovery and neuronal protection. Furthermore, EpoD treatment reduced not only the tau pathology, but also, and more interestingly and novel, the presynaptic and extracellular accumulation of Aβ. Thus, MT-stabilizing compounds should be considered as therapeutic candidates to modify AD progression, targeting both Aβ and tau pathology.

## Materials and methods

### Transgenic mice and Epothilone D treatment

Generation and characterization of APP^751SL^/PS1^M146L^ (APP/PS1) has been previously reported^24,28,31,32,34,35,71^. These APP/PS1 mice were obtained by crossing heterozygotic Thy1-APP^751SL^ (Swedish-K670N, M671L- and London-V717I-FAD mutations) mice with homozygous PS1^M146L^ mice (Charles River, France). Male and female 3-month-old APP/PS1 mice were randomly assigned to two groups to receive weekly intraperitoneal injections of 2 mg/kg Epothilone D (EpoD; Med. Chem. Express, USA) (APP/PS1^EpoD^, n = 8) or vehicle solution (APP/PS1^Veh^, n = 7) over a period of 3 months. Vehicle solution consisted of 1:1 (v/v) mixture DMSO/saline (NaCl) to reduce DMSO toxicity. Animals were fed ad libitum with standard mice diet (2014 Teklad Global 14% Protein Rodent Maintenance Diet, Harlan, Spain). Signs of abnormal behavior, distress, and body weight were weekly monitored. The EpoD treatment was well tolerated and no significant decrease in survival rate or weight was detected. All animal experiments were performed in accordance with the Spanish and the European Union regulations (RD53/2013 and 2010/63/UE) and approved by the Animal Research Committee from the University of Malaga (Spain). Experiments and procedures with animals were designed to minimize animal suffering and reduce the number of animals used.

### Behavioral studies

During the last 2 weeks of the treatment, 6-month-old APP/PS1^EpoD^ (n = 8) and APP/PS1^Veh^ (n = 7) mice were submitted to motor function and cognitive tests. Non-transgenic wild-type (WT; n = 12) mice of the same genetic background and age were also included. Behavioral tests were performed during the light period of the light/dark cycle and the experimenter was blind to the genotypes and treatment of mice. Habituation was performed as described earlier^30^. All behavioral registrations were performed using the monitoring software Ethovision XT 7.0 (Noldus, Netherlands).

*Open-field test* (OFT) was used to examine motor function^30,72^ by means of spontaneous locomotor activity and vertical exploratory behavior or rearings. Mice were placed in a square-shape arena (30 × 30 × 15 cm) and allowed to explore for 5 min. The locomotor activity was measured as the distance travelled and navigation speed with the software, while vertical exploratory behavior was quantified using an observational method.

### Object recognition test

The object location task (OLT) and the novel object recognition task (NORT) were used to evaluate hippocampus-dependent spatial and non-spatial memory, respectively^73,74^. Both tests were performed 1 and 24 h after the habituation to the open-field. Both tasks consisted of an acquisition and a discrimination phase (test phase), of 10 min each one, with a delay of 3 h between them. During OLT, two identical objects were presented to the animals and in the test phase, one of the objects was displaced to a novel location. For NORT, mice are allowed to explore two identical sample objects. Then, one of the familiar objects was replaced by a novel one. The basal measure was the time spent by the mice exploring objects during the sample phases and the test trial. Additionally, two discrimination indexes were calculated for the test trial: a location recognition ratio (total time exploring displaced object/total time of exploration) and an object recognition ratio (total time exploring novel object/total time of exploration). The time was recorded only when the mice touched the object with their nose.

### Y-maze specific context

This test was used to evaluate the long-term memory considering how familiar the animal finds a specific context^75,76^. The maze consists of three acrylic arms (31 × 18 × 32 cm) placed at 120° respect to each other, with different internal visual clues. Animals performed two trials (training and test), with a 24 h inter-trial interval. During training, one arm was blocked off with a panel (novel arm). The mice were placed and allowed to freely explore the other two arms for 8 min. In the test trial, all arms were accessible and mice explore freely for 5 min. Time spent by the animal in each arm, distance (cm), and speed (cm/s) were analyzed.

### Morris water maze (MWM)

This test evaluates spatial cognition and memory^77,78^ using a circular pool (1.4 m diameter, San Diego Instruments, Inc., California, USA), where animals have to locate a hidden platform beneath opaque water, guided by some spatial cues^79,80^. Mice were trained to find the platform (learning or acquisition phase) for 7 days (4 trials/day, maximum trial duration 90 s, intertrial interval of 5 min). On the day 7, 1.5 h after the last trial, the platform was removed and animals were allowed to explore the maze for 60 s (retention phase). To discard visual or motivational deficiencies, on day 8 animals performed the visible platform test (4 trials, maximum trial duration 60 s). Latency, distance travelled, speed, and time spent in the target quadrant (retention phase) were analyzed.

### Tissue preparation

Three days after the last day of the treatment, mice were anesthetized with sodium pentobarbital (60 mg/kg) and transcardially perfused with 0.1 M phosphate buffered saline (PBS). Then, brain was quickly removed; left hippocampus was dissected out and frozen while right hemisphere was fixed by immersion with 4% paraformaldehyde, 75 mM lysine, 10 mM sodium metaperiodate in 0.1 M phosphate buffer (PB), pH 7.4 for 5 days at 4 °C. Fixed hemispheres were cryoprotected in 30% sucrose, coronally sectioned at 40 μm thickness in a freezing microtome and serially collected in cold PBS and 0.02% sodium azide.

### Antibodies

The following primary antibodies and dilutions were used: anti-Aβ42 rabbit polyclonal (1:5,000, Abcam); anti-Aβ (clone 6E10) mouse monoclonal (1:5,000, Signet); anti-human Aβ, N-terminus (clone 82E1) mouse monoclonal (1:6,000, IBL); anti-acetylated α-tubulin (clone 6-11B-1) mouse monoclonal (1:50,000, Sigma); anti-human amyloid precursor protein (hAPP) rabbit polyclonal (1:20,000, Sigma); anti-ATP synthase β (clone 10/ATP) mouse monoclonal (1:10,000, Brand BD Transduction Laboratories); anti-β-Actin (clone AC 74) mouse monoclonal (1:10,000, Sigma-Aldrich); anti-phospho-PHF-tau pSer202/Thr205 (clone AT8) mouse monoclonal (IHC 1:1,000, Cell Signaling); anti-PSD95 rabbit monoclonal (1:2,000, Cell signaling); anti-Somatostatin (SOM) goat polyclonal (1:1,000, Santa Cruz Biotechnology); anti-synaptophysin rabbit polyclonal (1:1,000, Abcam); anti-total tau (TAU46) mouse monoclonal (1:1,000, Cell Signaling) and anti-ubiquitin rabbit polyclonal (1:5,000, Dako).

### Total protein extraction and Western blots

Total protein was extracted as described previously^29,34,71,81^. Protein pellets from dissected hippocampi, obtained using the Tripure TM Isolation Reagent, were resuspended in 4% SDS and 8 M urea in 40 mM Tris–HCl, pH 7.4 and rotated overnight at room temperature. Western blots (WB) were performed as described previously^82^. Briefly, 7–15 µg of protein from the different samples were loaded on 10% and 12% SDS-Tris–Glycine-PAGE and transferred to nitrocellulose (Hybond-C Extra, Amersham, Sweden). To analyze Aβ, protein samples were loaded onto 16% SDS-Tris-Tricine-PAGE and transferred to PVDF (Inmobilon-P, Millipore). After blocking, using 5% non-fat milk, the membranes were incubated overnight, at 4 °C, with the appropriate antibody. Membranes were then incubated with the corresponding horseradish-peroxidase (HRP)-conjugated secondary antibody (Cell Signaling) at a dilution of 1:10,000. Each blot was developed using the ECL-plus detection method (Amersham, Sweden). For quantification, images were obtained using Image-Quant Las 4000 mini gold (GE Healthcare Bio-Sciences) and analyzed using PCBAS program. In each experiment, the intensity of bands from WT mice and/or experimental condition were averaged and considered as 100% relative units. For normalization purposes, proteins were first estimated by Lowry and protein loading corrected by beta-actin. Data were always normalized by the specific signal observed in 6-month-old WT group.

### Synaptosomes isolation

The synaptosomal fractions were obtained as described previously^28^. Briefly, the tissue was homogenized using a Dounce homogenizer in 0.32 M Sucrose, 10 mM Tris–HCl (pH 7.4) buffer (buffer A) containing complete protease and phosphatase inhibitor cocktails (Sigma). After homogenization, the crude synaptosomal fraction (synaptosomes plus mitochondria) was isolated by two sequential centrifugations (1,500×*g*, 10 min followed by 12,500×*g*, 20 min; at 4 °C). The crude synaptosomes were resuspended in 13% (final concentration) Ficoll 400 (in buffer A) and layered on the bottom of a discontinuous gradient, composed by buffer A and 7% Ficoll (in buffer A). The gradients were centrifuged at 100,000×*g* (45 min at 4 °C) and the synaptosomes were isolated at the 7.5–13% interface. After washing (twice with buffer A), the protein content of the synaptosomal fractions was quantified by Lowry.

### BACE-1 and γ-secretase activity determination

BACE-1 activity was determined using a commercial kit (R&D Systems, Germany) following the manufacturer instructions. Briefly, fresh hippocampal membranes were solubilized in the buffer supplied by the manufacturer, centrifuged at 10,000×*g* (15 min at 4 °C) and the supernatant (100–200 μg of protein) was used for BACE-1 assay. The γ-secretase activity was determined as described previously^29^**.** Briefly, membranes, isolated from APP/PS1 cortex, were resuspended (at 3 mg of protein per ml) in 150 mM Citrate Buffer, pH 6.4, containing protease inhibitors (Roche). Aliquots (150 μg of proteins) were used for each assay. Samples were then incubated, at 37 °C with orbital shaking, for 2 h in absence or in presence of Epothilone D (100 nM). After incubation, membranes were sonicated (at 80 W for 30 s) and centrifuged at 30,000×*g* (30 min, 4 °C). Supernatants were used to determine AICD production by western blot using anti-APP C-terminal antibody. As negative control, membranes were keep on ice (4 °C) and treated as above.

### APPswe-expressing N2a cultures

APPswe-stably transfected Neuroblastoma cells were generously donated by Dr. Gopal Thinakaran (University of Chicago).

N2aAPPswe cells were cultured as described^29^. For EpoD treatment, a 0.2 μm filtered stock solution of Epothilone D was diluted in the same media at a final concentration of 100 nM. This media was kept for 12 or 24 h before collecting the cells and isolating RNA and protein as described above.

### Immunohistochemistry

The immunolabeling procedures were done as described earlier^28–34^. Serial sections from all animals were processed in parallel for immunostaining using the same batches of solutions to minimize variability during immunostaining processing. Free-floating sections were first treated with 3% H_2_O_2_/10% methanol in PBS, pH 7.4 for 20 min to inhibit endogenous peroxidases, and with avidin–biotin Blocking Kit (Vector Labs, Burlingame, CA, USA) for 30 min to block endogenous avidin, biotin and biotin binding proteins. After primary antibody incubation (24 h at room temperature), sections were incubated with the corresponding biotinylated secondary antibody (1:500 dilution, Vector Laboratories) followed by streptavidin conjugated HRP (1:2000, Sigma-Aldrich). The peroxidase reaction was visualized with 0.02% 3-3-diaminobenzidine tetrahydrochloride (DAB, Sigma-Aldrich), 0.03% nickel ammonium sulphate and 0.01% hydrogen peroxide in PBS. Sections immunolabeled for APP, AT8, ubiquitin or SOM were incubated in 0.2% Congo-red solution. For immunofluorescent labeling, sections were incubated with the primary antibody followed by Alexa 568 secondary antibody (1:1,000, Invitrogen). Specificity of the immune reactions was controlled by omitting the primary antisera. For Thioflavin-S staining, free-floating sections were incubated for 5 min with 0.015% Thio-S (Sigma) in 50% ethanol, and then rinsed in 50% ethanol and PBS. Quantitative comparisons were carried out on sections processed at the same time with same batches of solutions.

### Image analysis

*Plaque loading* (percentage of total CA1 area Aβ42-immunopositive or stained with Thioflavin-S), *dystrophic neurite loading* (percentage of total CA1 area covered by APP-positive or Ubiquitin-positive dystrophic neurites) and *Synaptophysin-positive area* (ratio of immunopositive area of the molecular sublayer/molecular layer area) were quantified. All image quantification was performed as previously reported^30,31,33,35^. Immunostained sections (4 sections/animal) from APP/PS1^EpoD^ (n = 7) and APP/PS1^Veh^ (n = 4) mice were visualized under a Nikon Eclipse 50i microscope coupled with a Nikon DS-5M high-resolution digital camera. ACT-2U program (Auto Camera Tame to You, Imaging software, Nikon Corporation 2004) was used to take digital images of hippocampal regions using a 10 × objective. Thioflavin-S staining was examined under an Olympus BX-61 epifluorescent microscope using FITC filter and 4 × objective. Images were acquired with an Olympus DP71 high-resolution digital camera using the Cell-A program (Olympus). The camera settings were adjusted at the start of the experiments and maintained for uniformity. Digital images were analyzed using Visilog 6.3 analysis program (Noesis, France). Staining densities were identified by level threshold, which were maintained throughout the experiment for consistency. Grey-scale images were converted to binary images. The sums were averaged and a single plaque burden was computed for each mouse.

The CA1 area or dentate gyrus molecular layers in each image was manually outlined and in the case for dystrophic neurite loading the positive somata were removed by manual editing.

### Stereological analysis

#### Neuron and dystrophic neurites counting

SOM-positive cells and SOM- or AT8-positive dystrophic neurites were stereologically quantified in the CA1 subfield of APP/PS1^EpoD^ (n = 7) and APP/PS1^Veh^ (n = 4) groups according to the optical fractionator method as described previously^30,31,33–35^. Wild-type mice (n = 4) were used as control group for SOM-cell counting. Briefly, the quantitative analyses were performed using an Olympus BX61 microscope interfaced with a computer and an Olympus DP71 digital camera, and the NewCAST (Computer Assisted Stereological Toolbox) software package (Olympus, Denmark). Cell/dystrophic quantification was done through the rostrocaudal extent of the hippocampus (between − 0.94 mm anterior and 3.64 mm posterior to Bregman coordinates), in every seventh section (with a distance of 280 μm), and an average of 7 sections were analyzed for each animal. CA1 boundaries were defined using a 4 × objective and the number of neurons was counted using a 100 ×/1.35 objective. We used a counting frame of 1874.2 μm^2^ with step lengths of 68.45 × 68.45 μm. The numerical density (ND; SOM cells or dystrophies/mm^3^) of immunopositive events was estimated using the following formula: ND = N/(A × 10_m/SV), where N is the number of dissector-counted somatic/dystrophic profiles, A, the area and SV is the volumetric shrinkage factor of the sample (the SV was determined in the same way as described by^34^. The precision of the individual estimations is expressed by the coefficient of error (CE). Total CE (CE group value) was calculated using the CEs in each individual animal. An investigator who was blinded to the experimental conditions performed neuronal/dystrophic profile counts.

#### Plaque size

Plaque morphometric analysis was performed as previously reported^30^ using the nucleator method with isotropic probes by the NewCAST software package from Olympus stereological system. CA1 subfield was analyzed in sections from APP/PS1^Veh^ (n = 4) and APP/PS1^EpoD^ (n = 7) mice immunostained with anti-Aβ42 using a counting frame of 6,022.8 μm^2^. For individual plaque measurement, a 40 × objective was used. Each analysis was done by a single examiner blinded to sample identities.

### Statistical analysis

Statistical analyzes were performed using the GraphPad software 7.0. Data are presented as mean ± standard deviation (SD), except for the cognitive data expressed as mean ± standard error of the mean (SEM). The comparison between two groups was done by two-tailed Student’s *t* test. To compare several groups, we used one-way ANOVA and one-way or two-way ANOVA with repeated measures (RM) followed by Tukey post-hoc test. The significance was set at 95% of confidence.

## Supplementary information

Supplementary Figures.

## Data Availability

All data generated in this study are included in this article and its online supplementary information.
