# Supplementary material for: Enhancing microtubule stabilization rescues cognitive deficits and ameliorates pathological phenotype in an amyloidogenic Alzheimer’s disease model
Source: Sci Rep. 2020 Sep 8;10:14776. doi: 10.1038/s41598-020-71767-4 (PMC7479116; doi:10.1038/s41598-020-71767-4)
Supplement: Supplementary file 1 — Supplementary Figures. [file 41598_2020_71767_MOESM1_ESM.pdf]

Supplementary Information for:

ENHANCING MICROTUBULE STABILIZATION RESCUES COGNITIVE DEFICITS AND AMELIORATES PATHOLOGICAL PHENOTYPE IN AN AMYLOIDOGENIC ALZHEIMER'S DISEASE MODEL

Juan Jose Fernandez-Valenzuela[1,2§], Raquel Sanchez-Varo[1,2§], Clara Muñoz-Castro[2,3,4], Vanessa De Castro[1], Elisabeth Sanchez-Mejias[1,2], Victoria Navarro[2,3,4], Sebastian Jimenez[2,3,4], Cristina Nuñez-Diaz[1,2], Angela Gomez-Arboledas[1,2], Ines Moreno-Gonzalez[1,2], Marisa Vizuete[2,3,4], Jose Carlos Davila[1,2], Javier Vitorica[2,3,4*], and Antonia Gutierrez[1,2*]

1-Dpto. Biología Celular, Genética y Fisiología, Instituto de Investigación Biomédica de Málaga-IBIMA, Facultad de Ciencias, Universidad de Málaga, Spain

2-Centro de Investigación Biomédica en Red sobre Enfermedades Neurodegenerativas (CIBERNED), Madrid, Spain

3- Dpto. Bioquímica y Biología Molecular, Facultad de Farmacia. Universidad de Sevilla, Spain

4-Instituto de Biomedicina de Sevilla (IBIS)-Hospital Universitario Virgen del Rocio/CSIC/Universidad de Sevilla, Spain

[§]Both contributed equally as first authors; * Co-senior authors

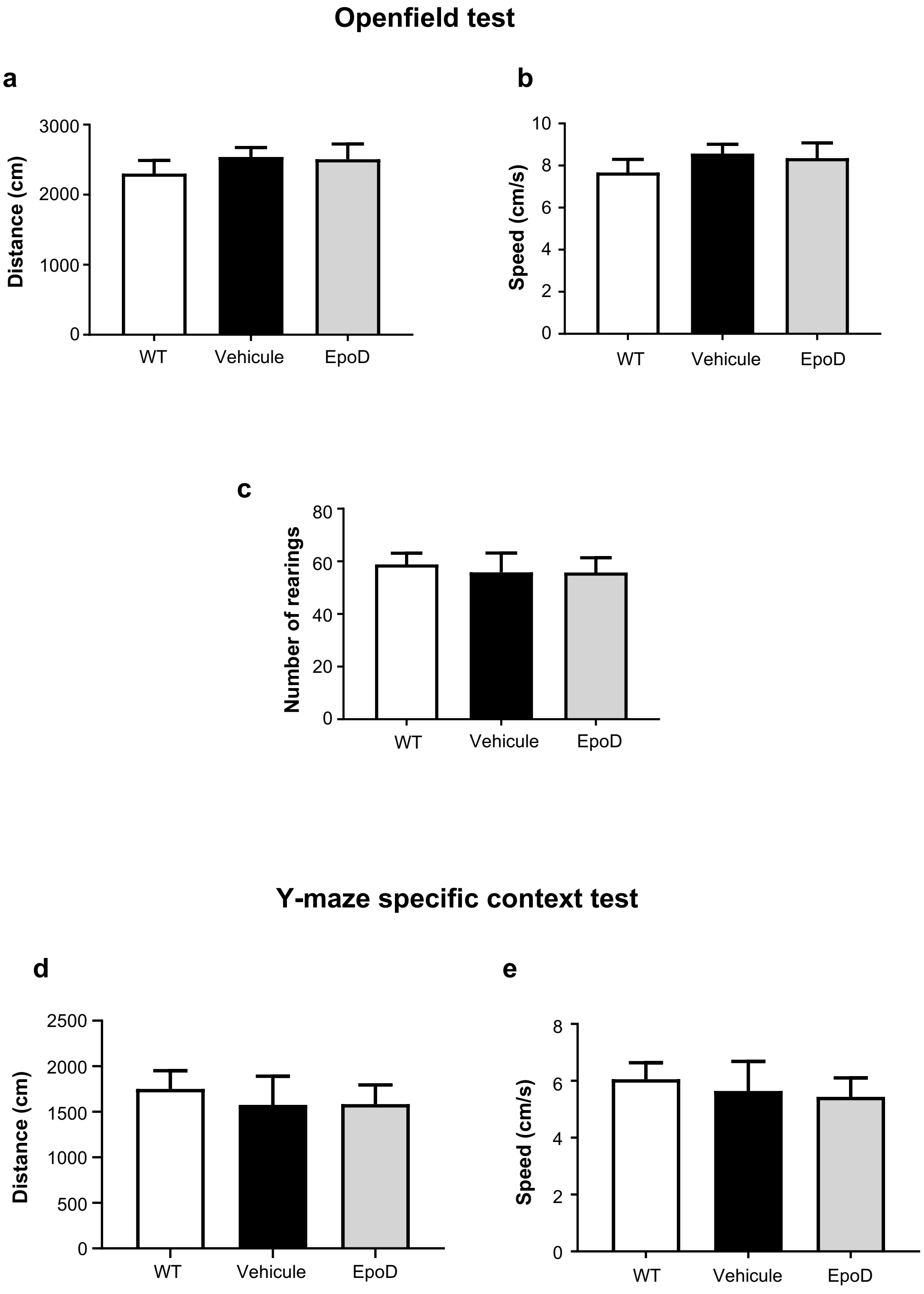

Supplementary Figure S1. EpoD treatment did not affect the locomotive or exploratory behavior of APP/PS1 mice. At openfield test, one-way ANOVA revealed no significant differences between groups in (a) the distance roamed (F (2,22)=0.491; p=0.619); (b) speed (F (2,22)=0.576; p=0.571) or (c) the number of rearings (Kruskal-Wallis test, p = 0.419). At Y-maze specific context test, one-way ANOVA showed no significant differences between groups in (d) distance roamed (F (2, 21)=1.529; p=0.2398) nor in (e) speed (F (2, 21)=1.628; p=0.2202). All data shown correspond to mean ± SEM.

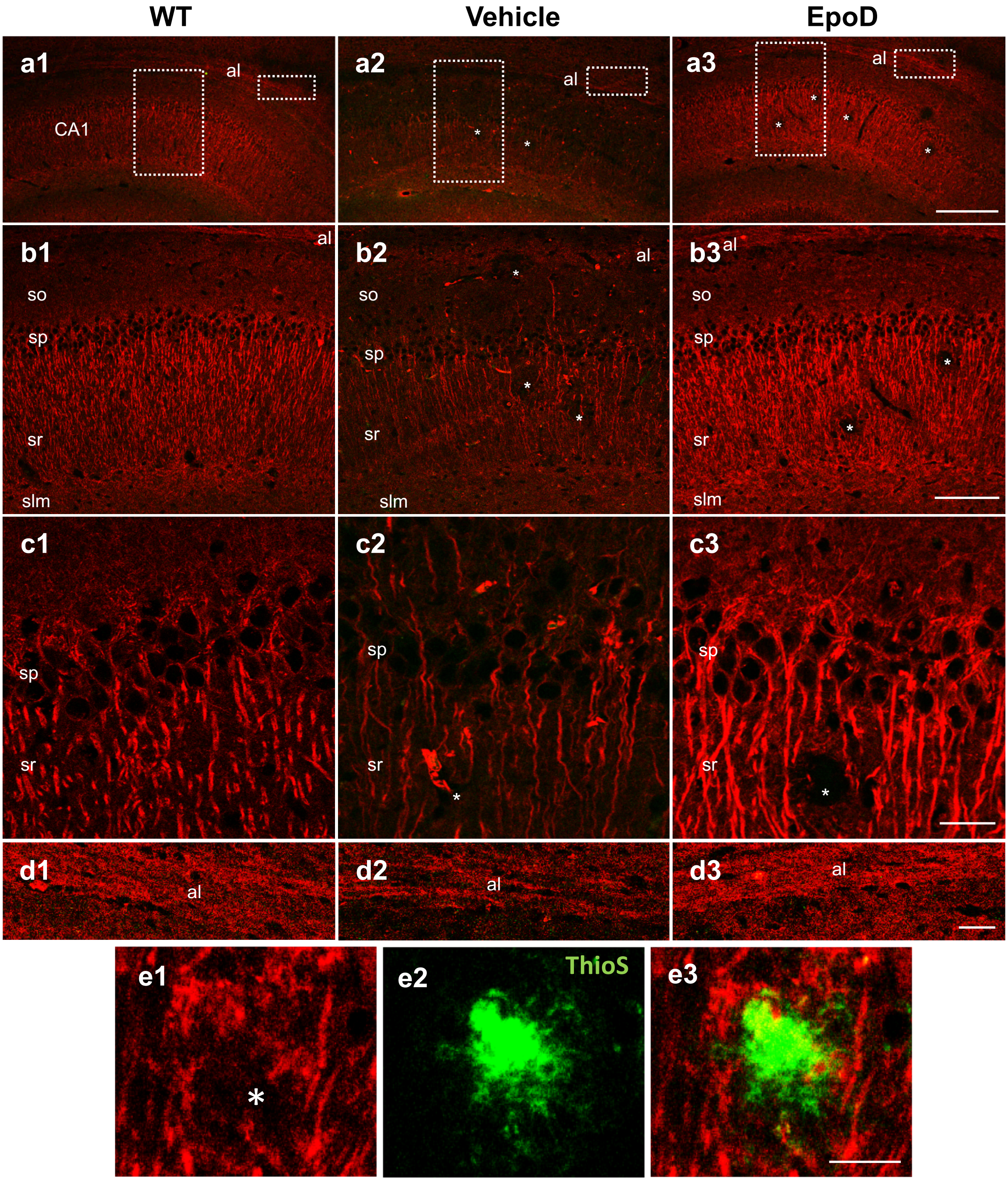

Supplementary Figure S2. Epothilone D treatment promoted microtubular stability in APP/PS1 hippocampus. Confocal images of acetylated-tubulin (AcTub) immunofluorescence in the hippocampal CA1 region of WT (a1-d1), APP/PS1^{Veh} (a2-d2) and APP/PS1^{EpoD} (a3-d3) mice. Higher magnification images of the alveus are shown (d1-d3). AcTub immunofluorescence combined with thioflavin-S staining in APP/PS1 mice (e1-e3) showing that area labeled with asterisks (in a2-c2 and a3-c3) are occupied by amyloid plaques. Al, alveus; so, stratum oriens; sp, stratum pyramidale; sr, stratum radiatum. ThioS, thioflavin-S. Scale bars: a, 250 μm; b-c, 100 μm; d, 25 μm; e, 15 μm.

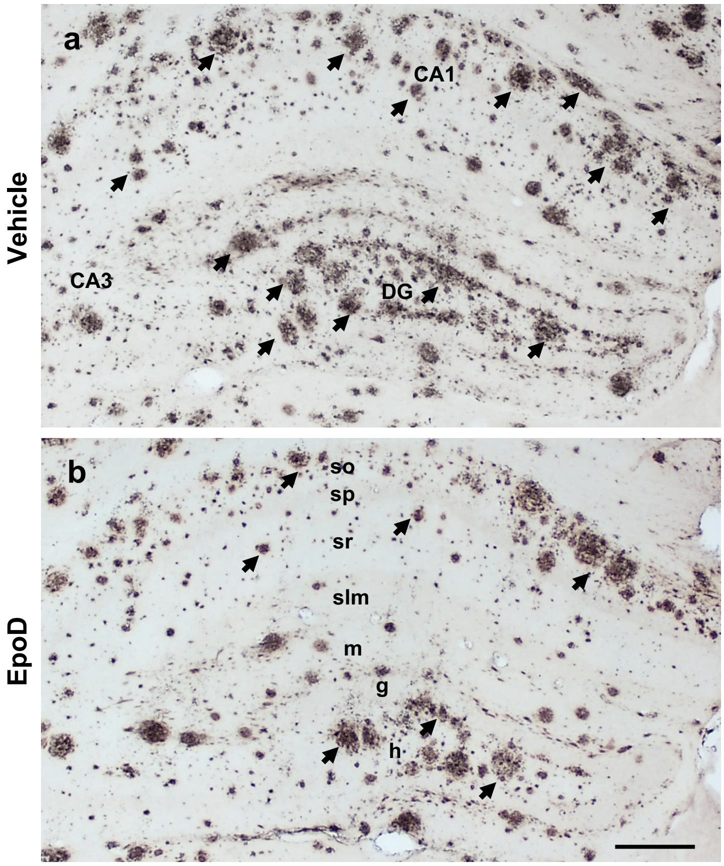

Supplementary Figure S3. Aβ42 immunostaining in the hippocampus of APP/PS1^{Veh} and APP/PS1^{EpoD}. Panoramic views of Aβ42 immunoreactivity in vehicle and EpoD-treated mice hippocampus. Extracellular Aβ42 accumulation was higher in the hippocampus proper and dentate gyrus from vehicle (a) than in the EpoD group (b). CA, Cornu ammonis; DG, dentate gyrus; So, stratum oriens; sp, stratum pyramidale; sr, stratum radiatum; slm, stratum lacunosum-moleculare, m, stratum moleculare, g, granular layer; h, hilus. Scale bar: a-b, 250 μm.

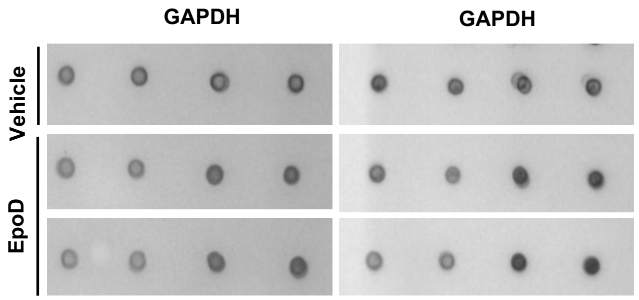

Supplementary Figure S4. Loading controls for S1 dot blot experiments. In parallel experiments to those showed in Fig 6c1, soluble S1 samples from Vehicle (n=4) or EpoD (n=8) treated animals were dotted and developed using anti-GAPDH antibody. These dot blots were quantified and used for OC and A11 normalization.

EpoD reduced Abeta production in N2a-APP cell line

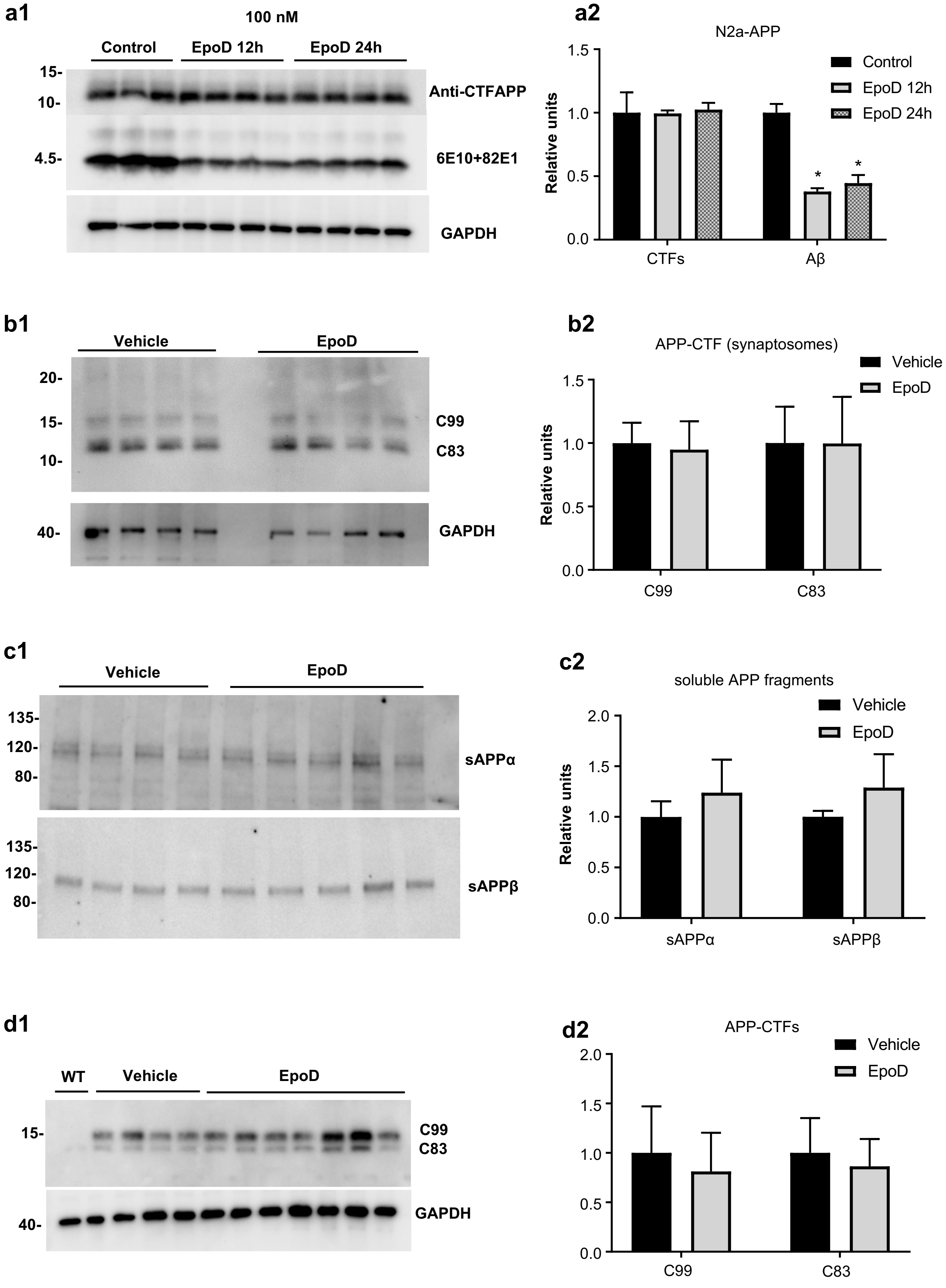

Supplementary Figure S5. EpoD effect on Aβ production and APP processing. (a1) N2a-APP cell line was used to test the effect of EpoD treatment (100nM during 12h or 24h) on APP-derived CTF fragments (upper western) or intracellular Aβ production (middle western). Parallel cultures were treated with vehicle (controls, n=3) of 100nM EpoD for 12h (n=4) or 24h (n=4). After incubation, cells were washed and proteins extracted and used for western blot analysis. (a2) Right panel showed the quantitative analysis of these experiments. (b1) Synaptosomal APP-CTFs (C99 and C83 fragments) were analyzed (by anti-APP C-terminal antibody) using proteins isolated from synaptosomal fractions obtained from Vehicle (n=4) or EpoD treated APP/PS1 mice. (b2) Right panel showed the quantitative analysis of these experiments. APP-derived soluble (c1) or CTFs (d1) fragments from Vehicle (n=4) or EpoD (n=5 for sAPP or n=7 for C99/C83 CTFs) treated APP/PS1 mice were analyzed using anti-APPα or APPβ specific antibodies or anti-APP-C-terminal antibody. (c2-d2) Quantitative analysis were showed in the right panels.

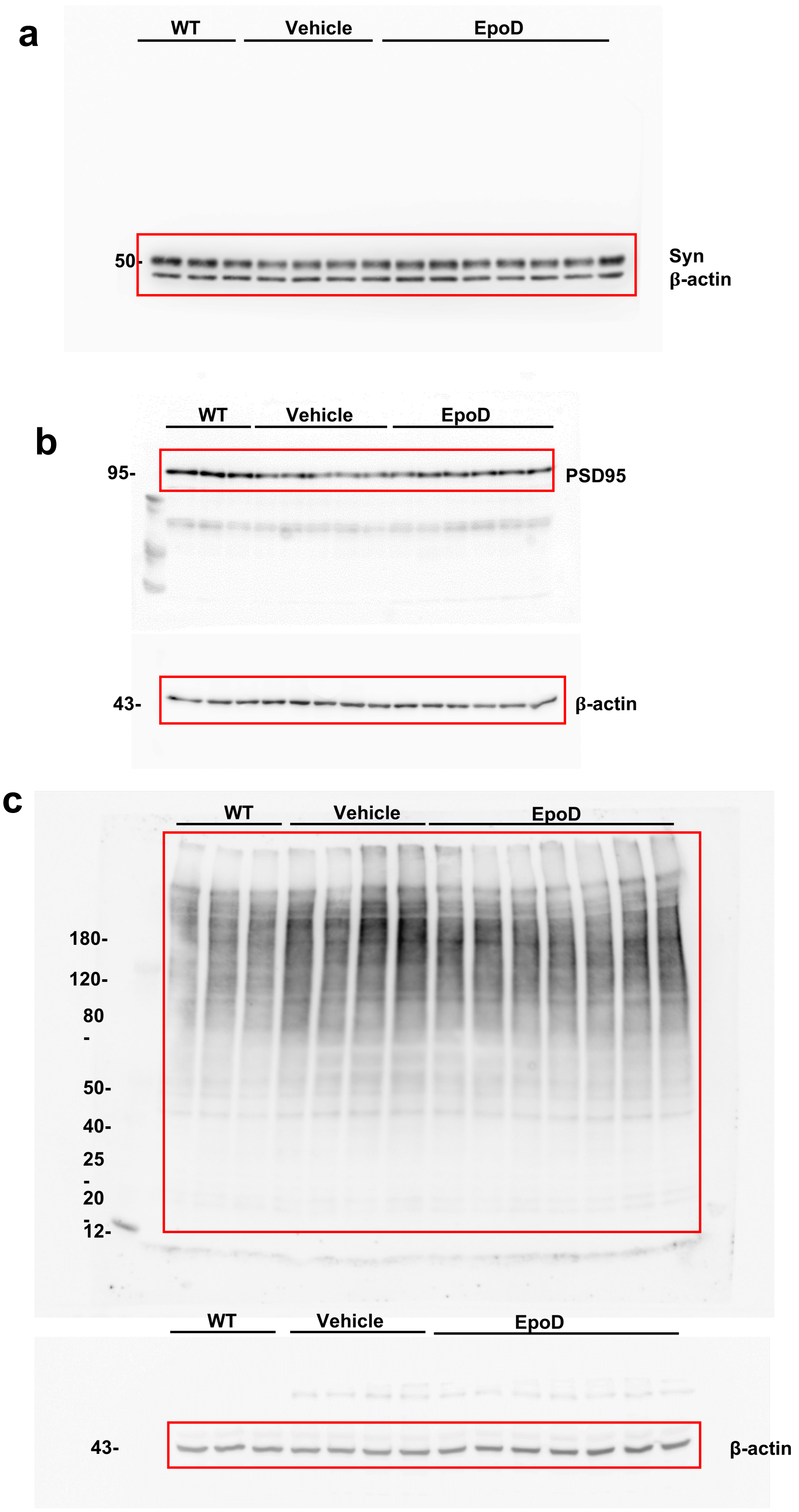

Supplementary Figure S6. Full length blots for Figure 2. (a) Synaptophysin and β-actin. (b) PSD95 and β–actin. (c) Ubiquitin and β–actin. Cropped areas in Fig. 2a, 2e and 2g are shown in red.

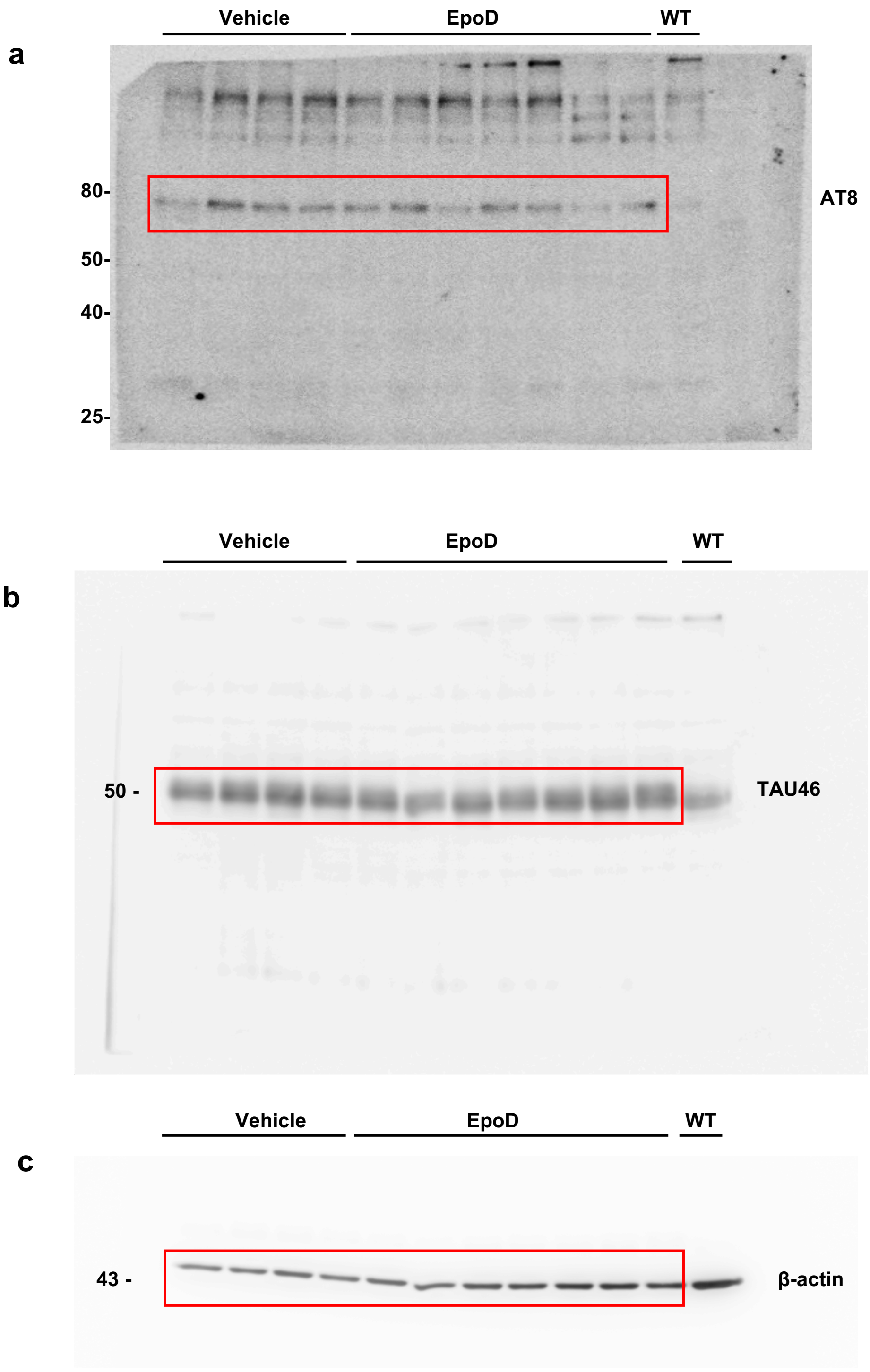

Supplementary Figure S7. Full length blots for Figure 4. (a) AT8 (phospho-tau), (b) TAU46 (tau total) and (c) β-actin. Cropped areas in Fig. 4a are shown in red.

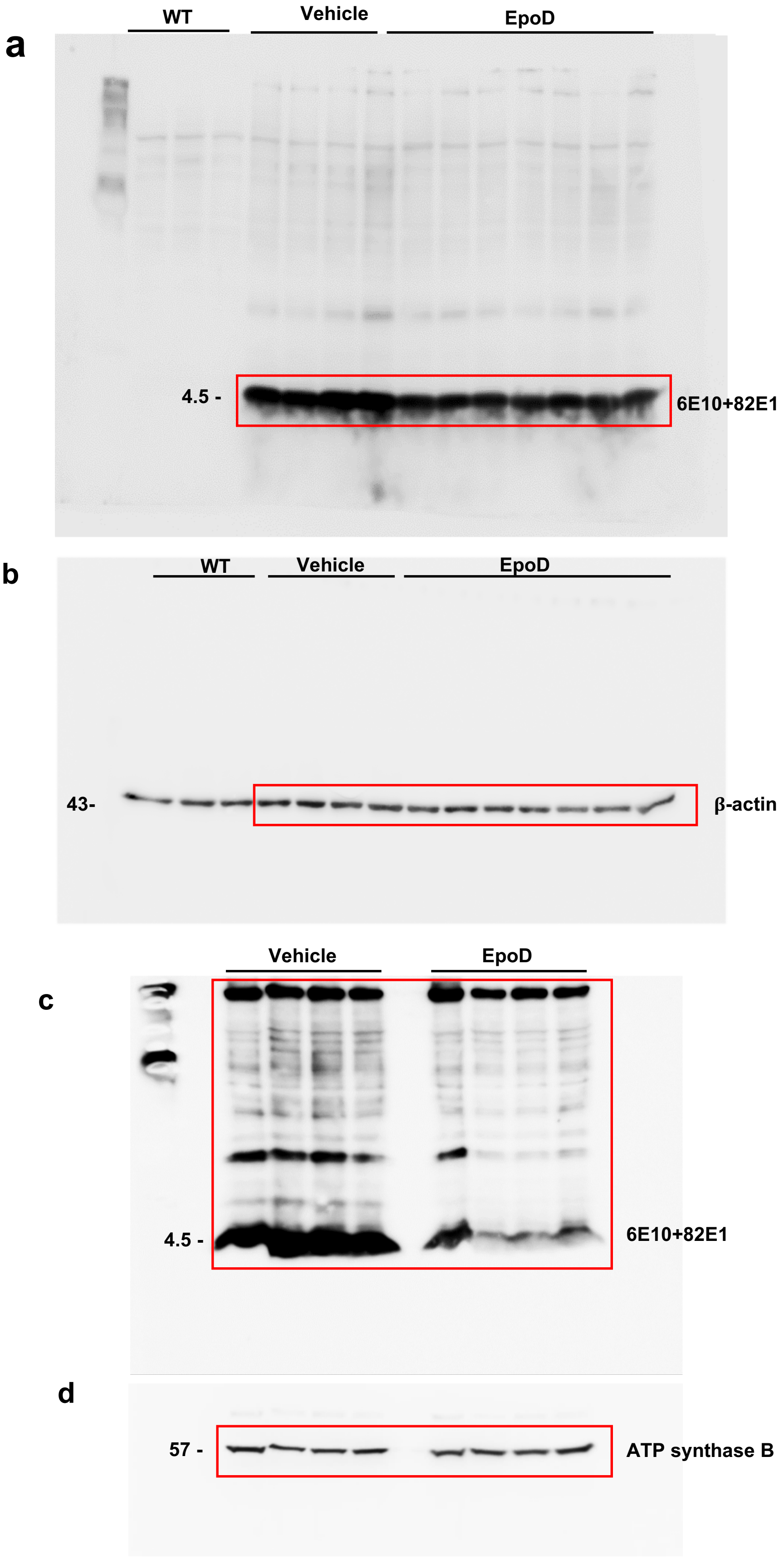

Supplementary Figure S8. Full length blots for Figure 6 a1 and b1. (a) Hippocampal total monomeric Aβ (6E10 plus 82E1) and β–actin. (b) Synaptosomal monomeric Aβ (6E10 plus 82E1) and (c) ATP-synthase B. Cropped areas in Fig. 6a1 and 6b1 are show in red.

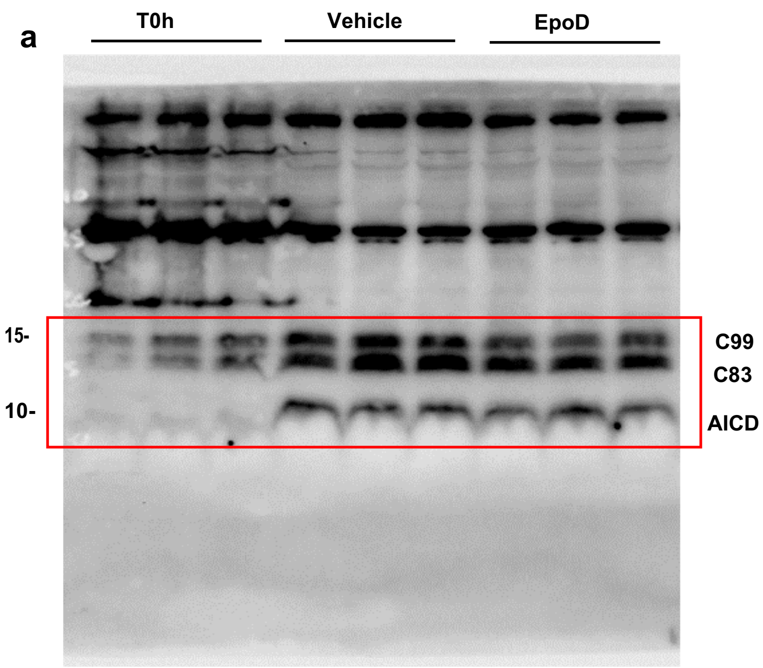

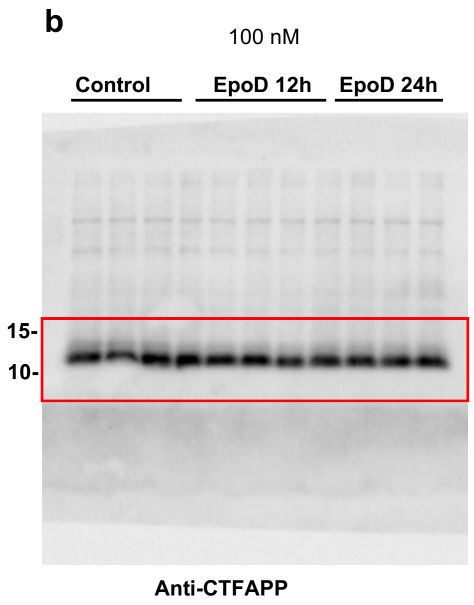

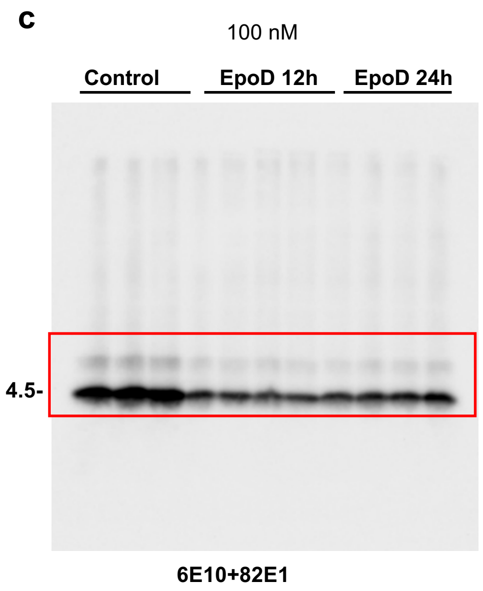

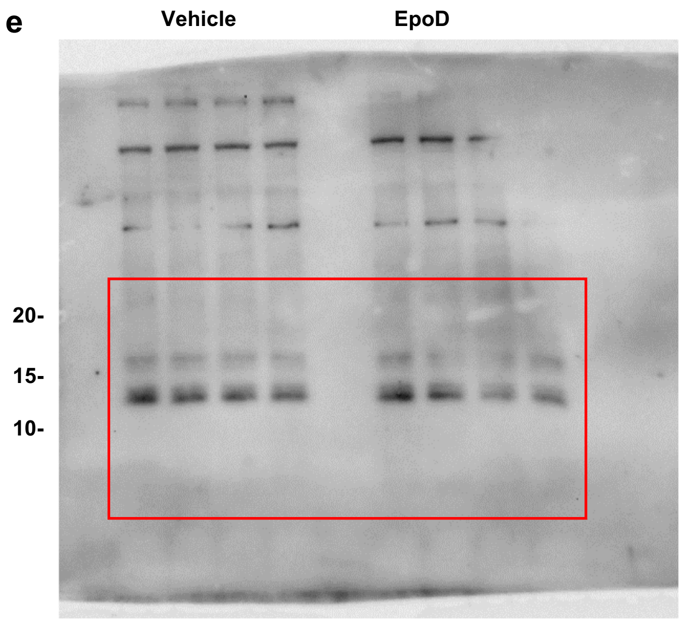

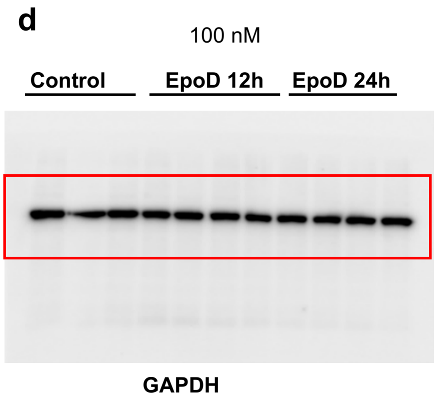

Supplementary Figure S9. Full length blots for Figure 6e1 and supplemental Fig. S5 a and b. a) Gamma-secretase activity. Cropped area in Fig. S6e1 is shown in red. b-d) Uncropped gels corresponding to Supplemental Fig. S5 a and b.

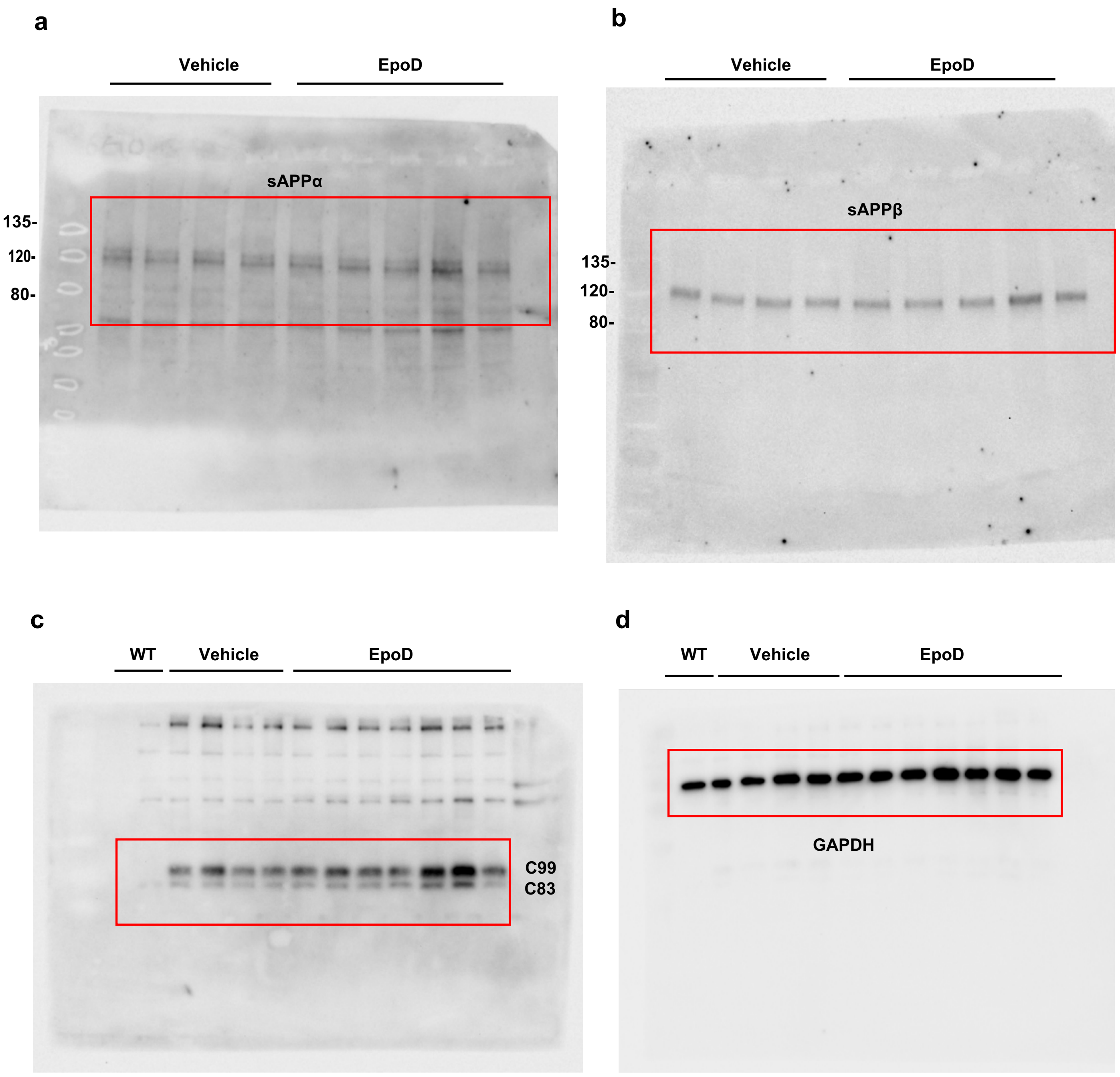

Supplementary Figure S10. Full length blots for supplemental Fig. 5 c1 and d1. Uncropped western blots corresponding to Fig. S5 c1 and d1. Cropped area are shown in red.
